# Characterization of *Auxenochlorella protothecoides* acyltransferases and potential of their protein interactions to promote the enrichment of oleic acid

**DOI:** 10.1186/s13068-023-02318-y

**Published:** 2023-04-21

**Authors:** Kui Liu, Jinyu Li, Chao Xing, Hongli Yuan, Jinshui Yang

**Affiliations:** State Key Laboratory of Animal Biotech Breeding, College of Biological Sciences, China Agricultural University, Beijing, 100193 China

**Keywords:** Diacylglycerol acyltransferase, Functional conservation, *Auxenochlorellaprotothecoides*, Acyl-CoA binding protein, Protein interactome, Oleic acid

## Abstract

**Background:**

After centuries of heavy reliance on fossil fuel energy, the world suffers from an energy crisis and global warming, calling for carbon emission reduction and a transition to clean energy. Microalgae have attracted much attention as a potential feedstock for biofuel production due to their high triacylglycerol content and CO_2_ sequestration ability. Many diacylglycerol acyltransferases (DGAT) species have been characterized, which catalyze the final committed step in triacylglycerol biosynthesis. However, the detailed structure–function features of DGATs and the role of the interactions among DGAT proteins in lipid metabolism remained largely unknown.

**Results:**

In this study, the three characterized DGATs of *Auxenochlorella protothecoides* 2341 showed distinct structural and functional conservation. Functional complementation analyses showed that ApDGAT1 had higher activity than ApDGAT2b in yeast and model microalgae, and ApDGAT2a had no activity in yeast. The N-terminus was not essential to the catalysis function of ApDGAT1 but was crucial to ApDGAT2b as its enzyme activity was sensitive to any N-terminus modifications. Similarly, when acyl-CoA binding proteins (ACBPs) were fused to the N-terminus of ApDGAT1 and ApDGAT2b, zero and significant activity changes were observed, respectively. Interestingly, the ApACBP3 + ApDGAT1 variant contributed to higher oil accumulation than the original DGAT1, and ApACBP1 + ApDGAT1 fusion boosted oleic acid content in yeast. Overexpression of the three DGATs and the variation of ApACBP3 + ApDGAT1 increased the content of C18:1 of *Chlamydomonas reinhardtii* CC-5235. Significantly, ApDGAT1 interacted with itself, ApDGAT2b, and ApACBP1, which indicated that these three lipid metabolic proteins might have been a part of a dynamic protein interactome that facilitated the enrichment of oleic acid.

**Conclusions:**

This study provided new insights into the functional and structural characteristics of DGATs and elucidated the importance of these physical interactions in potential lipid channeling.

**Supplementary Information:**

The online version contains supplementary material available at 10.1186/s13068-023-02318-y.

## Background

The continuous energy consumption and ballooning population have resulted in rising demands for biofuel. Triacylglycerols (TAGs) are the primary storage lipids in various eukaryotes [1]. They are energy reservoirs and signal molecules in many biological processes [2]. With substantial TAGs accumulation ability under adverse environmental conditions and the advantage of avoiding competition with food crops for arable land [3], microalgae have great nutritional and industrial value, especially as a promising renewable feedstock for biodiesel production [3, 4].

Diacylglycerol acyltransferase (DGAT) catalyzes the last step in de novo TAG synthesis in higher plants and microalgae [5]. To date, three isoforms of DGATs have been identified, which could be designated type-1 DGAT (DGAT1), type-2 DGAT (DGAT2 and DGTT), cytoplasmic DGAT3 (cytoDGAT). In addition to these three DGATs, bifunctional wax ester synthetase also had DGAT enzyme activity [6–8]. DGAT1 and DGAT2 in plants and microalgae have been extensively studied, especially in the TAG manipulation and oil production of different organisms [9–12]. Noticeably, the roles of DGAT1 and DGAT2 in lipid synthesis vary from species to species. For instance, in plants, DGAT1 is considered a significant player in seed oil accumulation for some oil crops [13, 14]. At the same time DGAT2 is vital in incorporating unusual fatty acids into storage TAG in plants [15, 16]. Similarly, DGAT1s and DGAT2s play different crucial roles in TAG biosynthesis in other microalgae [9, 10, 17, 18], but the contribution of different DGAT genes to TAG biosynthesis remains poorly understood.

DGAT1 and DGAT2 are both integral membrane proteins of the endoplasmic reticulum (ER), but possess a different number of transmembrane domains (TMDs) in the membrane topology [19, 20]. Several structure–function features of DGAT1 and DGAT2 have been characterized in mice, plants, and yeast. DGAT1 possesses a variable hydrophilic N-terminus that might have distinct functions and also a conserved C-terminus region with eight to ten TMDs [5]. In *Brassica napus*, the N-terminus domain of DGAT1 was not essential to DGAT activity but contributed to modulating activity at acyl-CoA/CoA levels [21]. Furthermore, BnaDGAT1 and *Mus musculus* DGAT1 were shown to self-associate and interact with acyl-CoA through positive cooperativity [22, 23]. The N-terminus of microalgae *Chromochloris zofingiensis* DGAT1 had a similar structure–function relationship with plant DGATs, and the fusion of an acyl-CoA binding protein (AtACBP6) to the N-terminus of CzDGAT1 successfully improves the DGAT activity [19]. The removal of two TMDs from Murine DGAT2 disrupted the ER localization, but the enzymatic activity of DGAT2 in promoting TAG storage was not affected [24]. However, deleting the first TMD of ScDGAT2 did not affect its association with microsomal membranes but resulted in the total loss of DGAT activity in *Saccharomyces cerevisiae*. In addition, the enzymatic activity was sensitive to modifications in both the hydrophilic N- and C-terminus of ScDGAT2, indicating the structural importance of these two terminus [25]. Similar results have also been reported in *C. zofingiensis* DGAT2s [20]. However, in other words, the TMDs and N-terminus domains of DGATs play essential roles in modulating activity. Although a growing number of DGATs have been characterized, the detailed structure–function information of DGATs and the effects of modulation on improving DGAT performance in microalgae were yet to be explored in depth.

In recent years, self-interaction among DGAT1 and DGAT2 has been reported, which might form homodimer or homotetramer, regulating DGAT activity and promoting TAG synthesis [22, 26–28]. Furthermore, direct interactions among lipid metabolic enzymes have also been observed in plants, including the interactions between ACBP2 and lysophospholipase 2 in *Arabidopsis* [29], the interactions between sn-glycerol-3-phosphate acyltransferase (GPAT) 8 and DGAT2 or GPAT9 in *Vernicia fordii* [30] and flax (*Linum usitatissimum*), the self-interactions between DGAT1, PCAT2 and PDCT1 [28], suggesting that lipid metabolic enzymes might have formed a dynamic protein interactome that facilitated triacylglycerol formation and fatty acid metabolism[28, 31]. In microalgae, interactions were only observed in CzDGAT1 and CzDGAT2s. It remains to be determined whether there is a similar dynamic protein interactome in the lipid metabolic enzymes and what physiological roles protein–protein interaction plays in microalgae.

The green microalgae *Auxenochlorella protothecoides (A. protothecoides)*, previously known as *Chlorella protothecoides*, is a typical oleaginous microalga with commercially important value [32, 33]. It has excellent potential to serve as a food and energy source because of its high photosynthetic efficiency and ability to accumulate up to 70% neutral lipids [33]. Moreover, a high proportion of 48% of *A. protothecoides* was oleic acid (C18:1), which makes it a suitable feedstock for biodiesel production and various industrial applications [34]. Although the physiological and biochemical characteristics of *A. protothecoides* has been extensively studied, its molecular mechanism contributing to lipid biosynthesis and TAG accumulation has yet to be thoroughly investigated.

Previous transcriptome studies had annotated three putative DGAT-encoding genes in oleaginous microalgae *A. protothecoides* UTEX 2341, namely ApDGAT1, ApDGAT2a, and ApDGAT2b [35]. This study characterized the functions and interactions of ApDGATs and ApACBPs in *A. protothecoides* UTEX 2341 and discovered that ApDGAT1 played a more critical role in TAG synthesis than ApDGAT2b. Notably, the structure–function features of the N-terminus of ApDGAT1 were distinctively different from that of ApDGAT2b. ApDGAT1 also interacted with itself, ApDGAT2b, and ApACBP1. It was proposed that microalgae lipid metabolic enzymes might have been a part of a dynamic protein interactome that facilitated TAG synthesis. This study would clarify the microalgae lipid accumulation mechanism and provide new ideas for obtaining desired microalgae oil products through genetic engineering and targeted transformation of microalgae lipid components.

### Methods

## Algal strains and growth conditions

*A. protothecoides* UTEX 2341 in this study was cultured on the following three media: nitrogen-replete heterotrophic medium (LM) [35], nitrogen-deficient medium (LM-N, i.e. LM without acid-hydrolyzed casein and ammonium chloride) and Cd stressed medium (LM-Cd, LM with 3 mM Cd added). The seed solution of *A. protothecoides* UTEX 2341 was cultured in LM with the same culture conditions as those described by Xing et al. [35]. Then, the cells were transferred to the above three media. For nitrogen deprivation experiments, the algae were collected by centrifugation at 8000 rpm for 5 min after 4 days of culture in LM, washed twice with LM-N, and then transferred to LM-N for another three days of culture. After cultivation, algal cells were collected by centrifugation, washed with deionized water twice, and weighed after freeze-drying. And the dry weight and oil content of the algae were calculated.

*Chlamydomonas reinhardtii* strains CC-5235 gifted by Jin Liu [36] were grown on a TAP medium under a light density of 30 μmol m^−2^ s^−1^ at 25 °C in an orbital shaker at a speed of 150 rpm.

### RNA isolation, reverse transcription PCR, and quantitative real-time PCR (qPCR)

The methods of algal RNA extraction, quantification, genome digestion, and reverse transcription of RNA and RT-qPCR described by Xing et al. [35] were used at different time points. The primers for the genes quantified by qPCR were shown in Additional file 2: Table S1. All tests were performed in three biological and three technical replicates.

### Cloning of DGATs and ACBP genes

ApDGATs and ApACBP genes were PCR amplified using cDNA from culture in LM. Primers were designed to amplify the full-length DGATs and ACBPs according to the mRNA sequences in NCBI (Additional file 2: Table S2). PrimeSTAR® HS DNA Polymerase (Takara, China) was used. The 20-μL amplification system consisted of 10 μL of buffer, 1.6 μL of dNTPs, 0.5 μL of each primer, 0.3 μL of cDNA, 0.2 μL of polymerase and water. The amplification program was as follows: 10 s at 98 ℃ and 30 s at 68 ℃ for 2 min. Thirty cycles were conducted. The amplification products detected electrophoretically were recovered with a DNA gel recovery kit (Tiangen, China) and ligated to the M5 Hiper pTOPO-blunt simple vector (Mei5 Biotechnology, Co., Ltd., China). Positive clones were identified by PCR and screened by sequencing (Beijing Liuhe BGI Technology Co. Ltd).

### Bioinformatics and phylogenetic analyses of acyltransferase genes

The open reading frames (ORFs) of the four genes were analyzed by ORFinder (https://www.ncbi.nlm.nih.gov/orffinder). The conserved domain was analyzed by CD-search in NCBI (https://www.ncbi.nlm.nih.gov/Structure/cdd/wrpsb.cgi). Signal 5.0 and TMHMM were used to predict the signaling peptide and transmembrane domains, respectively. Phylogenetic analyses of DGATs from plants, animals, and microalgae were performed using MEGA6, with ClustalW used for sequence alignment. Using the neighbor-joining tree method, the Poisson model and complete deletion were chosen, and the operation was performed with a bootstrap value of 1000. Protein sequence details were shown in Additional file 2: Table S3. Similarly, the phylogenetic analyses of forty-three DGAT1 sequences were performed separately, and the details were shown in Additional file 2: Table S4.

### Plasmid construction and transformation of *S. cerevisiae* yeast cells

The coding sequences of ApDGATs, different truncated mutants of ApDGAT1, ApDGAT2b (Additional file 2: Table S5), and the fusion proteins ApACBPs + ApDGAT1/2b were constructed by PCR or overlap-PCR, and subsequently re-cloned into the pYES2/CT vector and transformed into yeast strains of H1246 and INVSc1, respectively, using the PEG/lithium acetate method [37]. H1246 and INVSc1 cells harboring the empty vector pYES2/CT (EV control) were used as negative and positive controls, respectively. The selection of yeast transformants and culture of positive colonies was performed according to Zhang et al. [37], with slight modifications: Transformants were grown in SC/uracil medium containing 2% glucose for 24 h, harvested by centrifugation at 4000×*g* for 1 min, and then resuspended in SC/uracil medium containing 2% galactose at an initial OD_600_ of 0.2 for gene expression induction, and cultures were grown at 30 ℃ for 96 h for further lipid analysis.

### Heterologous expression of the* A. protothecoides *UTEX 2341 genes in *C. reinhardtii*

The cDNAs encoding ApDGATs and ApACBP3 + ApDGAT1 were subcloned into the vector pOpt_Clover_Hyg [38]. *C. reinhardtii* CC-5235 cells were transformed with XbaI-linearized vectors using the glass beads method [39]. Algal transformants were selected on TAP plates with 15 μg mL^−1^ hygromycin. The integration of ApDGATs and ApACBP3 + ApDGAT1 genes into the genome was verified by genomic PCR.

### Analytical methods

The method for algal lipids extraction was the same as that described by Xing et al. [35]. The same method for yeast oil extraction as described by Zhang et al. [37] was used, with a slight modification: the extraction process was conducted twice, and then the chloroform phases were pooled. After drying with nitrogen, the oil was weighed and dissolved in chloroform. The lipids were further separated on silica gel 60 plates (Merck) with *n*-hexane: methyl tert-butyl ether: acetic acid (80:20:2 [v/v/v]) as the development solvent, and lipids were identified based on comigration with known TAG standards (Sigma). Lipids on the TLC plates were visualized by an imager after spraying with 8% phosphoric acid and 10% CuSO_4_. The TAG spots were quantified using ImageJ software.

For quantification, fatty acid methyl esters (FAMEs) were prepared by acid-catalyzed transmethylation and then analyzed by GC (Agilent Technologies, Wilmington, DE) equipped with a capillary DB-23 column (65.0 m × 0.25 mm × 0.25 μm) as described previously [36]. The total FA content was quantified using Methyl-nonadecanoate, C19:0 (Sigma) as an internal standard. The neutral lipids of algae and yeast were stained with BODIPY ® 505/515 and then observed by laser scanning confocal microscopy.

### Subcellular localization analysis of acyltransferases in tobacco leaves

The primers used for cloning ApDGAT1 and ApDGAT2b into the pSuper-1300-GFP vector are shown in Additional file 2: Table S2. The recombinant vectors were transferred into Agrobacterium GV3101. The Agrobacterium transformants harboring pSuper-1300-GFP-ApDGAT1 and pSuper-1300-GFP-ApDGAT2b were injected into the leaves of 4–5 week-old *Nicotiana benthamiana*. The fluorescence of the lower epidermis of leaves after infiltration for 2 days was visualized using a fluorescent microscope. The positive control was the ER marker gene with mCherry-ER, and the negative control was the blank plasmid pSuper-1300-GFP.

### Yeast two-hybrid (Y2H) screening

The cDNA encoding each ApDGAT1 and ApDGAT2b enzyme was ligated to the pGADT7 (AD) and pGBDT7 (BD) C-terminus fragments, respectively. The recombinant vectors of AD-ApDGAT1, BD-ApDGAT1, AD-ApDGAT2b, BD-ApDGAT2b, and AD-ApACBPs, BD-ApACBPs in pairs were transformed into the yeast strain AH109. The positive strains with each bait/prey combination expressed were screened and cultured on SD agar plates lacking Leu and Trp (SD-L–T). Then, the interaction was assayed on SD agar plates lacking Leu, Trp, and His (SD-L–T–H) plates after 1:10 serial dilution of yeast cells starting at an A_600_ value of 0.2. Rad53/Mck1 and empty bait/prey were used as the positive and negative controls. 3-Aminotriazole was used to inhibit self-activation.

### Bimolecular fluorescence complementation (BiFC) assays

The full-length ApDGAT1, ApDGAT2b, and ApACBPs genes were ligated with the vectors pSPYNE (YNE) and pSPYCE (YCE), respectively. YNE-Gus and YCE-Gus were used as the negative controls. The recombinant vectors of YNE/YCE-ApDGAT1/ApDGAT2b/ApACBPs were transferred into *Agrobacterium tumefaciens*, and then, the positive transformants were used to infect 4–5-weeks-old leaves of *N. benthamiana*. After 2 ~ 3 days, fluorescence was observed by laser scanning confocal microscopy. The empty bait/prey were used as the negative control.

### Statistical analysis

All experiments were performed using biological triplicates to ensure reproducibility. Values presented are means SDs. Statistical analyses were carried out using the SPSS statistical package. Differences were considered statistically significant at *p* values < 0.05.

## Results

### Phylogenetic analysis, protein structures, and membrane topology analysis of ApDGATs

Based on the annotated genomic sequence of *Chlorella protothecoides* [40], and our previous study of transcriptomes under low and high-temperature stress and Cd stress [35, 41], three putative DGAT gene sequences were identified, designated as ApDGAT1 (gene ID: F751_6502), ApDGAT2a (gene ID: F751_1386) and ApDGAT2b (gene ID: F751_0071) and cloned. The lengths of the cDNA, the encoded protein and the molecular mass of ApDGATs varied greatly, ranging from 1014 to 2145 bp, 337 to 714 amino acid residues, and 37.97 to 79.57 kDa, respectively. The theoretical isoelectric points of ApDGAT1, ApDGAT2a, and ApDGAT2b were 9.57, 9.42, and 9.56, respectively (Additional file 2: Table S6).

**Fig. 1:**
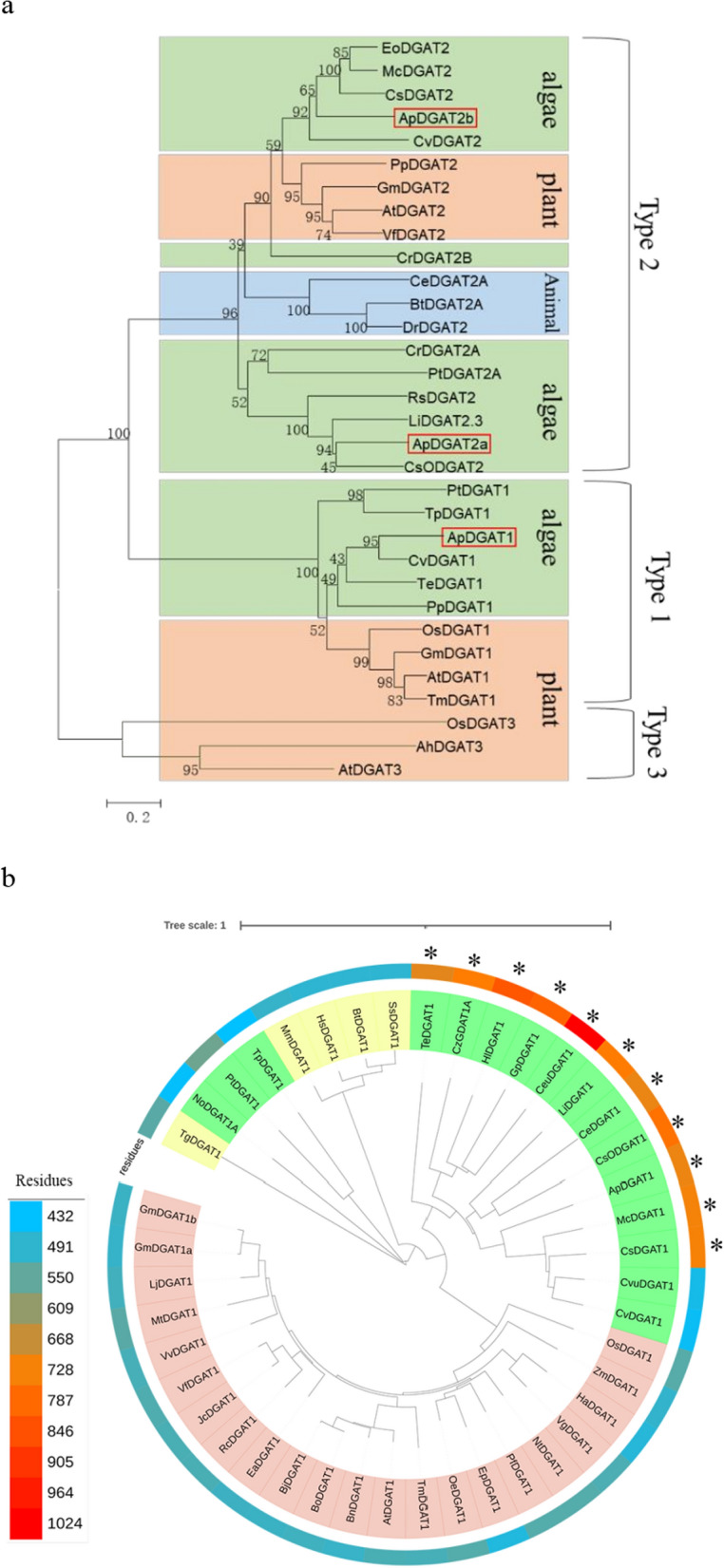
Cladogram of DGATs from plants, animals, and algae. **a** Cladogram of different types of DGATs. The pink, blue and green backgrounds represent plants, animals, and algae. **b** Cladogram of DGAT1 from forty-four sequences. The pink, yellow and green backgrounds represent plants, animals, and microalgae. The heatmap shows the amino acid residues of these DGAT1 sequences. '*' represents proteins with the PH domain

To examine the relationships among different sources of DGAT, phylogenetic analyses were performed on 45 DGATs from plants, animals, and microalgae. The results indicated that there were three subtypes of ApDGATs: ApDGAT1 (type-1 DGAT), ApDGAT2a, and ApDGAT2b (type-2 DGATs). Furthermore, ApDGAT1 and ApDGAT2 were found mainly clustered together with type-1 and type-2 DGATs derived from algae. Similarly, the DGATs from plants clustered together, as did the animal DGATs, which suggested that the evolution of DGATs was conserved mainly by vertical rather than horizontal transfer of genes (Fig. 1a). Further analysis of the forty-three type-1 DGAT sequences from plants, animals, and microalgae showed that only 11 DGAT proteins (marked "*") originated from microalgae contained pleckstrin homology (PH) domain (Fig. 1b), indicating that the PH domain might have played certain roles in DGAT activity.

The conserved domains with functional importance in ApDGATs were further analyzed (Additional file 1: Fig. S1). ApDGAT1 contained not only the MBOAT conserved domain but also a length of approximately 96 aa of the PH domain. ApDGAT2a and ApDGAT2b contained the LPLAT subfamily domain. ApDGAT2a contained a length of 19 aa signal peptides (SPs), while ApDGAT1 and ApDGAT2b had no SP (Additional file 1: Fig. S1). Moreover, ApDGAT1, ApDGAT2a, and ApDGAT2b contained 7, 11, and 2 TMDs, respectively (Additional file 1: Fig. S2). Intriguingly, previous studies showed that type-2 DGATs had less than 5 TMDs. The specific role of ApDGAT2a containing 11 TMDs needed to be further investigated.

### ApDGAT1 and ApDGAT2b were associated with TAG accumulation under nitrogen starvation and Cd stress in *A. protothecoides* UTEX 2341

**Fig. 2:**
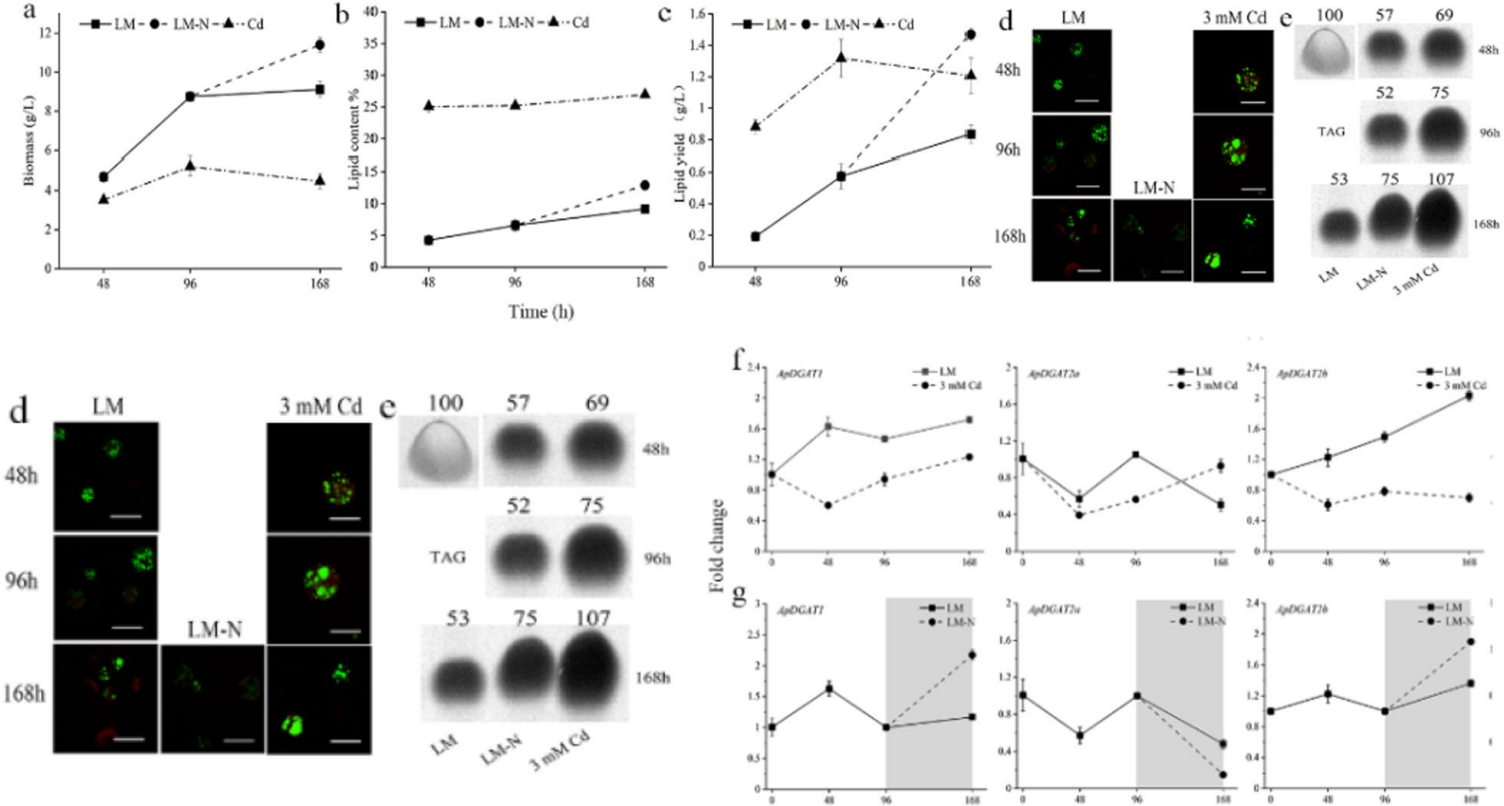
Growth, lipid variation, and ApDGATs expression of UTEX 2341 under heterotrophic condition (LM, as a control), LM-N, and LM-Cd. The square solid lines represent LM, the circular dotted lines represent LM-N, and the triangular dotted lines represent LM-Cd culture. The neutral lipids (TAGs) were qualitatively and quantitively analyzed by BODIPY ® 505/515 staining (**d**) and TLC (**e**), respectively. Relative TAG content was quantified by Image J software. The relative expression levels of ApDGAT1, ApDGAT2a, and ApDGAT2b in LM, LM-N, and LM-Cd were monitored by quantitative real-time PCR (qPCR) (**f**, **g**). Each sample was analyzed in biological and technical triplicates. Each data point represents the average of three biological replicates. The values represent the mean ± SD (*n* = 3)

To explore the roles of the three different ApDGATs in lipid accumulation, the mRNA expression patterns of the DGAT genes, the variation in algae cell biomass, and the total lipid and TAG content in heterotrophically fermented LM, LM-N and LM-Cd of *A. protothecoides* UTEX 2341 were investigated. As shown in Fig. 2a, the algal cell biomass in LM increased continuously to 9.1 g L^−1^ in the end. The lipid content and lipid yield in LM were ∼8.6% and 1.14 g L^−1^, respectively. The overall biomass level in LM-Cd was lower than that in LM and LM-N, reaching the highest of a mere 5.2 g L^−1^ at 96 h (Fig. 2a). In contrast, the lipid content was the highest in LM-Cd compared with those in LM-N and LM (Fig. 2b), but the lipid yield reached 1.47 g L^−1^ at 72 h in LM-N, higher than that in LM-Cd and LM (Fig. 2c). Moreover, both BODIPY® 505/515 staining (Fig. 2d) and TLC separation of total lipids (Fig. 2e) revealed that TAG largely accumulated under nitrogen starvation and Cd stress, with TAG content increased by 41.5% and 201%, respectively.

In addition, RT-qPCR was performed to examine the transcription patterns of ApDGATs in LM, LM-N, and LM-Cd (Fig. 2f, g). The expression levels of the three genes in different media varied with the extension of culture time. Compared with the stable expression levels in LM, nearly all three genes were decreased in expression profiles within 0–48 h in LM-Cd but continued to increase from 48 to 96 h, especially those of ApDGAT1 and ApDGAT2a, which continually increased till 168 h. Moreover, the expression levels of ApDGAT1 and ApDGAT2b at 72 h of nitrogen starvation were up-regulated by 86% and 39%, respectively. Nevertheless, the transcription of ApDGAT2a decreased significantly. This observation agreed with a previous study on *N. oceanica* and *C. zofingiensis* [10, 42]. Therefore, the upregulation of ApDGAT1 and ApDGAT2b was concomitant with the increase in the TAG and total lipid content under two abiotic stresses, suggesting that ApDGAT1 and ApDGAT2b might have played important roles in TAG accumulation.

### ApDGAT1 and ApDGAT2b recovered the TAG synthesis in the* S. cerevisiae* TAG-deficient mutant

**Fig. 3:**
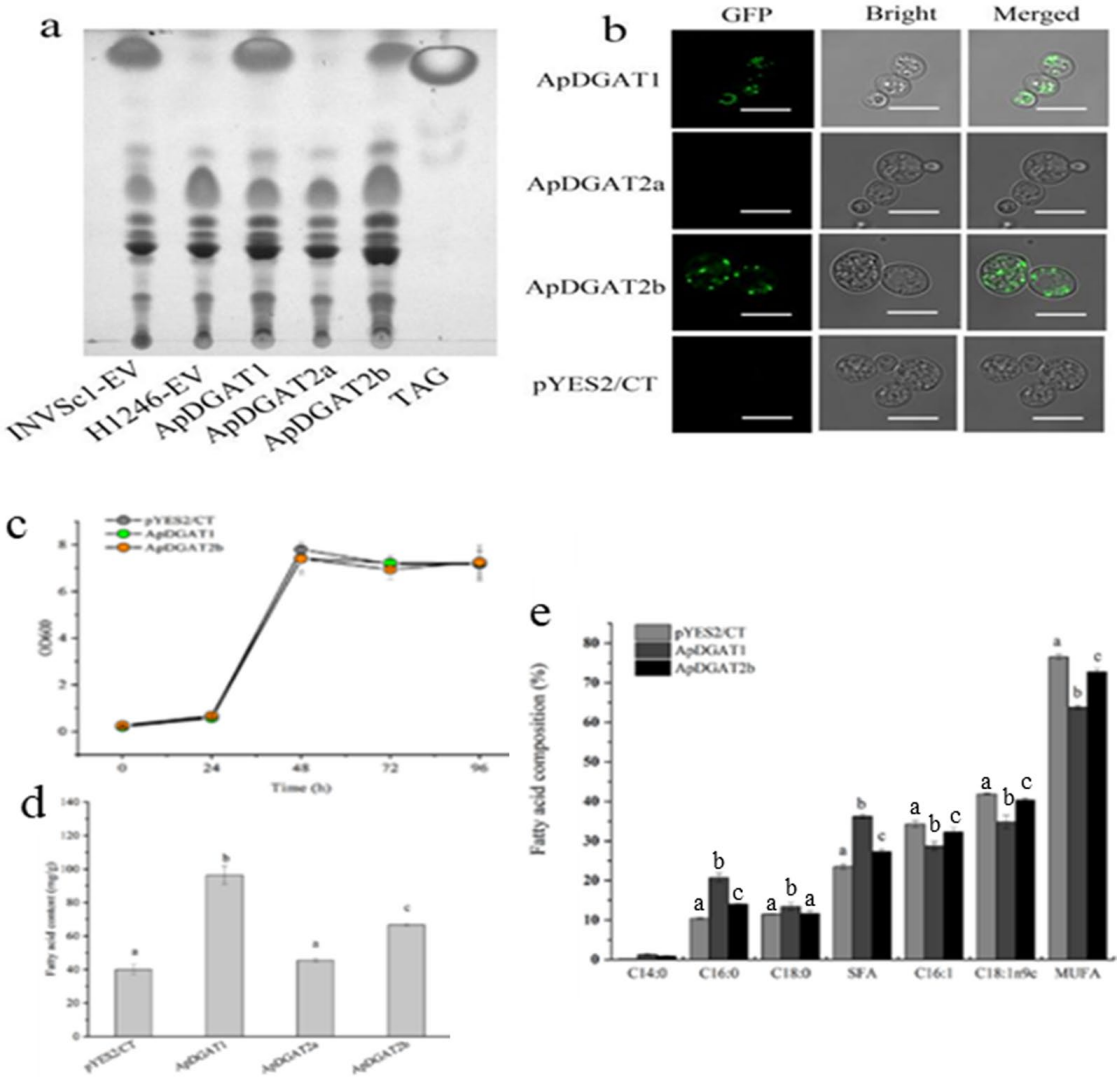
Functional complementation of acyltransferases in the TAG-deficient yeast strain H1246. **a** TLC analysis of lipids extracted from H1246 cells transformed with ApDGATs. INVSc1 and H1246 harboring the empty vector (EV) were used as positive and negative controls, respectively. **b** BODIPY staining of H1246 cells with ApDGATs expressed. Left, fluorescent field; middle, bright field; right, merged field. Green fluorescence indicates the BODIPY-bound lipid droplets. Bars = 7.5 µm. **c** The growth curve of H1246 transformed with EV, ApDGAT1, and ApDGAT2b. **d** The fatty acid content of H1246 transformed with EV, ApDGAT1, and ApDGAT2b. **e** Fatty acid composition of H1246 transformed with EV, ApDGAT1, and ApDGAT2b. Data are mean ± SD of three biological replicates. Different letters indicate significant differences at *p* < 0.05, as determined by one-way ANOVA with Tukey’s multiple comparisons test

To investigate whether putative ApDGATs could act as DGATs to catalyze TAG biosynthesis, they were heterologously expressed individually in TAG synthesis-deficient *S. cerevisiae* H1246 [42]. All transformants were harvested for TLC-based neutral lipid analysis (Fig. 3a) and BODIPY® 505/515 staining (Fig. 3b). Similar to the positive control, lipid bodies were present in the mutant strains transformed with the ApDGAT1 or ApDGAT2b, but were undetectable in the H1246 cells expressed with ApDGAT2a and the empty expression vector pYES2/CT (Fig. 3b). The total lipids were extracted from the transformants and then separated by TLC. Similarly, the results exhibited that TAG biosynthesis was restored in strains harboring ApDGAT1 or ApDGAT2b (Fig. 3a), which were consistent with the results from BODIPY® 505/515 staining (Fig. 3b). These results suggested that ApDGAT1 or ApDGAT2b could function as TAG synthases to catalyze the last step of TAG biosynthesis in the heterologous system. The activity of ApDGAT2b was found lower than that of ApDGAT1 as the fluorescence signal and TAG band from TLC analysis were weaker in ApDGAT2b than in ApDGAT1 of transgenic H1246.

To explore the effects of ApDGATs on the fatty acid profile in H1246, the total fatty acid content of yeast carrying ApDGAT1 or ApDGAT2b genes was measured by gas chromatography. With the strain growth unaffected (Fig. 3c), the fatty acid content of H1246 yeast strains transformed with ApDGAT1 or ApDGAT2b was 1.41 and 0.67 times higher than those of control, respectively (Fig. 3d). However, the total lipid content in H1246 yeast strains transformed with ApDGAT2a showed no significant difference from that of the negative control. Fatty acid profiles showed that levels of saturated fatty acyl-CoAs (SFAs, C16:0, and C18:0) were higher in both transgenic H1246 cells with overexpressed ApDGAT1 or ApDGAT2b than that in the H1246-EV control (Fig. 3e).

**Fig. 4:**
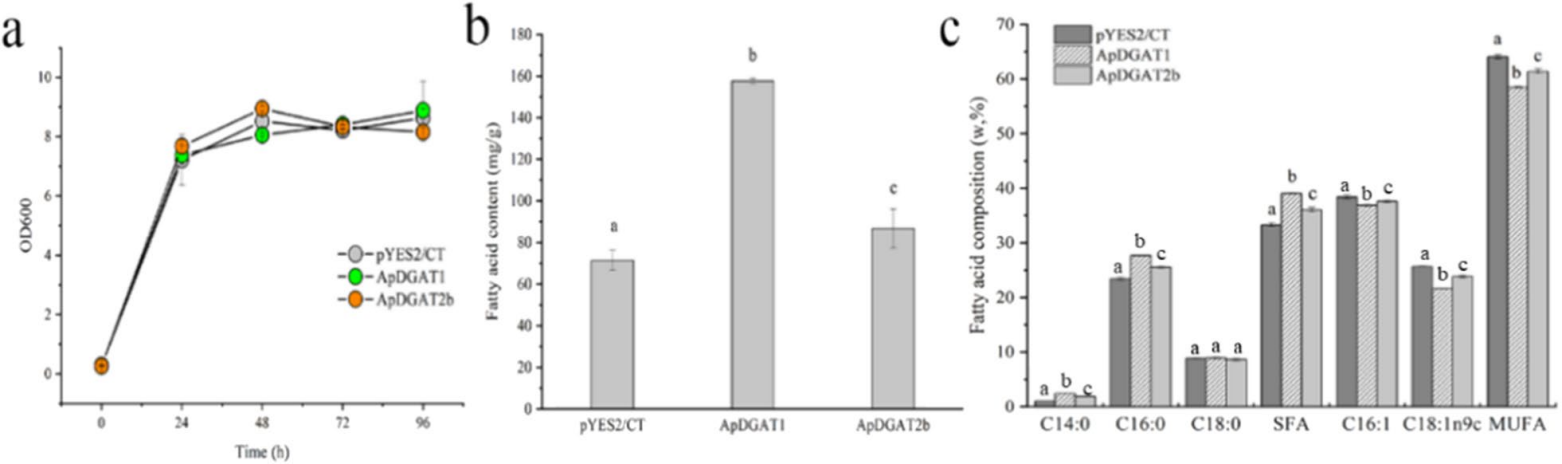
Overexpression of ApDGAT1 and ApDGAT2b in the yeast strain INVSc1. Comparison of **a** Growth, **b** Total fatty acid content and **c** Fatty acid composition between strains with ApDGAT1 and ApDGAT2b overexpressed and the EV control. Data are mean ± SD. of three biological replicates. Different letters indicate significant differences at *p* < 0.05, as determined by one-way ANOVA with Tukey’s multiple comparisons test

The functions of ApDGAT1 and ApDGAT2b were further verified in INVSc1, a yeast strain with a complete lipid synthesis pathway. Similarly, the strain growth was not affected by the expression of ApDGAT1 or ApDGAT2b (Fig. 4a). The total fatty acid content of INVSc1 transformed with ApDGAT1 and ApDGAT2b increased significantly by 2.2- and 1.21- fold, respectively (Fig. 4b). In addition, the fatty acid composition of INVSc1 transformed with ApDGAT1 or ApDGAT2b was similar to that of H1246 transformed with ApDGAT1 or ApDGAT2b, with the ratio of saturated fatty acid to unsaturated fatty acid increased in both transformants (Fig. 4c). The above functional verification showed that ApDGAT1 and ApDGAT2b played important roles in TAG synthesis, and both preferred to use saturated fatty acids in TAG synthesis and showed overlaps in substrate utilization, but ApDGAT1 contributed more to lipid accumulation in yeast than ApDGAT2b.

### Subcellular localization of ApDGAT1 and ApDGAT2b in tobacco leaves

**Fig. 5:**
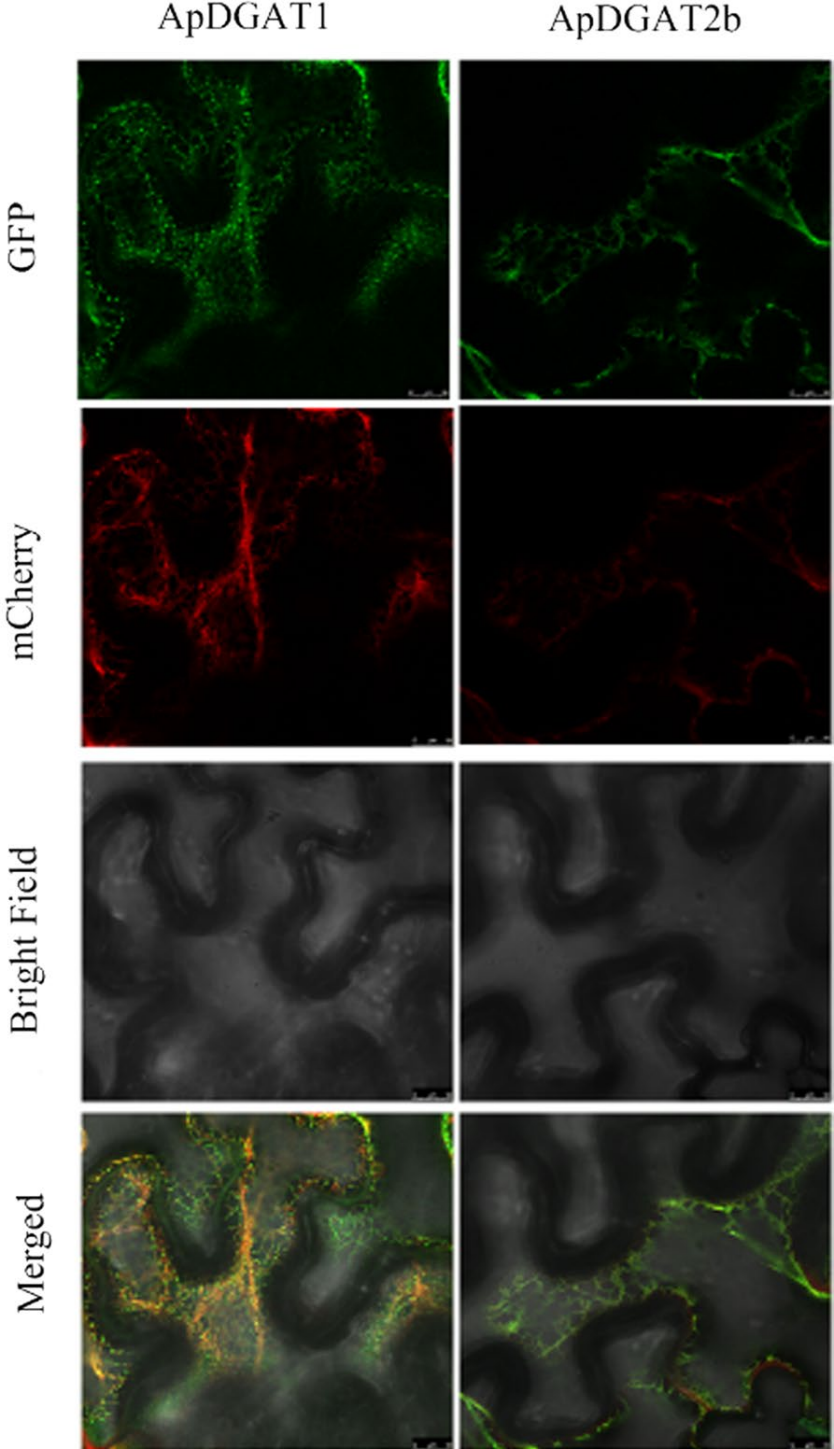
Subcellular locations of ApDGAT1 and ApDGAT2b in tobacco leaf cells. GFP represents the fluorescence produced by ApDGAT1 and ApDGAT2b. The red fluorescence of mCherry marks the endoplasmic reticulum (ER) position. Bright: bright field. Bars = 25 μm

Type-1 and type-2 DGAT are membrane-bound proteins and are considered to be endoplasmic reticulum localization proteins in higher plants and animals [43]. Silico assays detected several TMDs in ApDGAT1 and ApDGAT2b. To confirm whether ApDGAT1 and ApDGAT2b were located in ER, the proteins were tagged with GFP at the C-terminus and transiently co-expressed with the mCherry-tagged ER marker in leaves of *N. benthamiana*. The results showed that the green fluorescence of ApDGAT1 and ApDGAT2b overlapped with the red fluorescence of mCherry (Fig. 5), which indicated that both ApDGAT1 and ApDGAT2b were located in the ER.

### The distinct structure–function relationship between N-termini of ApDGAT1 and ApDGAT2b and DGAT enzymatic activity maintenance

**Fig. 6:**
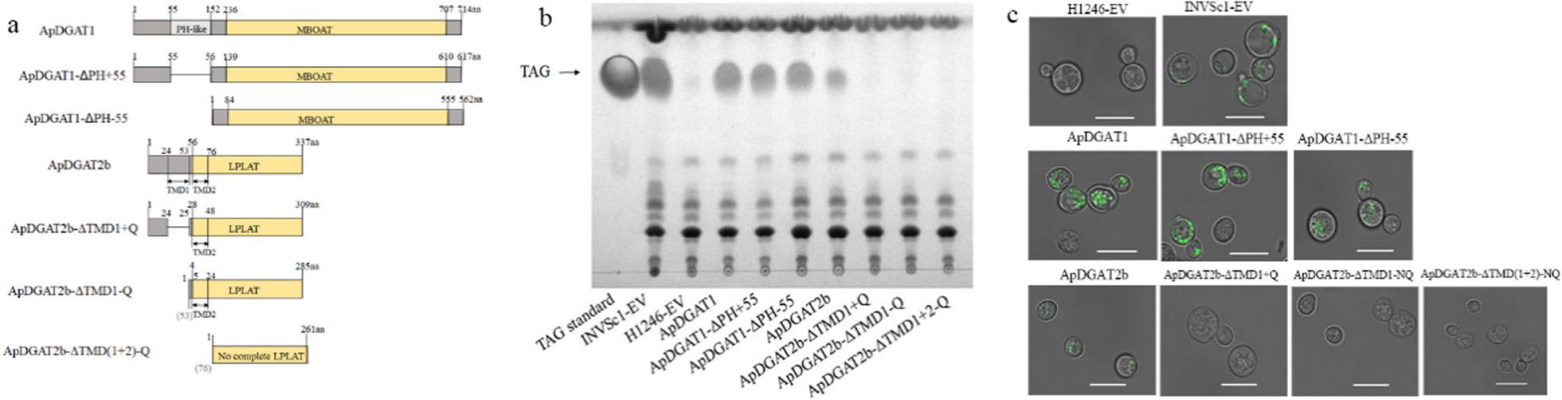
Functional complementation of different truncated proteins in the TAG-deficient yeast strain H1246. **a** Schematic representations of full-length ApDGAT1 and ApDGAT2b and their various mutants. **b** TLC analysis of the lipids extracted from H1246 cells transformed with truncated proteins. **c** BODIPY staining of H1246 cells expressed with truncated proteins. Green fluorescence indicates the BODIPY-bound lipid droplets. Bars = 7.5 µm

The N-terminus of ApDGAT1 was predicted to possess a special PH domain, which existed in type-1 DGAT1 of some microalgae. To probe the importance of the PH domain and N-terminus in enzymatic catalysis, full-length and various truncated proteins of ApDGAT1 were constructed (Fig. 6a), and their TAG synthesis ability was verified in yeast H1246. The results of PCR identification of transformants were shown in Additional file 1: Fig. S3. Interestingly, the TAG synthesis ability of ApDGAT1-ΔPH + 55 was not affected by deleting the PH domain according to the TLC analysis and the neutral lipid staining with BODIPY (Fig. 6b, c). These results indicated that the PH domain was dispensable to the enzyme activity of ApDGAT1. However, the removal of the PH domain from the protein of MiDGAT1 (LiDGAT1) in *Myrmecia incisa* led to weaker TAG synthesis ability [44], revealing that the PH domain of DGAT1 might have played different roles in modulating the DGAT activity in different microalgae.

Moreover, mutant ApDGAT1-ΔPH-55 exhibited lower DGAT activity than the full-length enzyme and mutant ApDGAT1-ΔPH + 55, according to the fluorescence signals and the TAG band from TLC analysis. Further N-terminus truncation of ApDGAT1-ΔPH-55 significantly decreased the TFA content, suggesting that the first 55 amino acids down-regulated enzymatic activity. These results revealed that the N-terminus domain of ApDGAT1 was dispensable to its enzyme activity maintenance, but distinct regions of this domain contributed differently to enzyme activity modulation.

ApDGAT1 and ApDGAT2b have distinct conserved domains and protein structures, and it is interesting to test whether the N-terminus of ApDGAT2b also serves a similar function in catalysis as the ApDGAT1. A previous study showed that after the deletion of two TMDs, murine DGAT2 was still active [24]. In this study, the function of two TMDs in the N-terminus of ApDGAT2b was first investigated. The schematic of these truncated proteins of ApDGAT2b was shown in Fig. 6a. The deletion of the two TMDs and N-terminus regions from the full-length ApDGAT2b led to complete enzyme inactivity and total TAG synthesis ability loss (Fig. 6b, c), indicating that both the N-terminus and TMDs domain of ApDGAT2b were indispensable to the enzyme activity of ApDGAT2b. These results suggested that the N-terminus domains of ApDGAT1 and ApDGAT2b differed in their importance in maintaining enzyme activity.

### Identification of putative ApACBPs and phylogenetic analysis and functional motif analysis of ApACBPs

Acyl-CoA-binding properties of the N-terminus region have been found in *Brassica napus*, mouse, and *C. zofingiensis* DGAT1s [19, 22, 23], and N-terminus fused with AtACBP6 kinetically improved CzDGAT1 enzyme activity [19]. In order to improve the enzyme activity of ApDGATs by increasing the acyl-CoA-binding properties, four putative ApACBPs were identified from previous transcriptome analyses [35]. Additional file 2: Table S2 listed the general information of the corresponding cDNAs and encoded enzymes.

Two important and conserved motifs previously identified, YKQA and KWDAW of ACBPs, were found acyl-CoA-binding and coenzyme-A head group-binding [45]. In this study, ACBP1 to ACBP4 were conserved at the acyl-CoA-binding domain (Additional file 1: Fig. S4a). As shown in Additional file 1: Fig. S4b, like other ACBPs, the four ApACBPs harboring two motifs (YKQA and KWDAW) showed remarkable sequence conservation, suggesting that ApACBPs might have played roles in acyl-CoA binding and transport. Phylogenetic analysis of protein sequences showed that ApACBPs could be classified into four groups. Specifically, ApACBP1 was grouped with *C. subellipsoidea* ACBP, *C. variabilis* ACBP, and *C. elegans* ACBP1, though the protein sequence similarity among them was less than 50.0%. ApACBP2 shared a respective sequence similarity of 55.0% and 57.8% with the algal group of *C. subellipsoidea* and *Trebouxia* ACBP. The sequence similarities between ApACBP3 and the group of *C. subellipsoidea*, *C. variabilis,* and *H. lacustris* ACBP were 67.7%, 52.3%, and 52.3%, respectively. ApACBP4 was clustered alone in the phylogenetic tree and far from the ACBP of plants, animals, or other algae (Additional file 1: Fig. S4c).

### Effects of fusing ApACBPs to the N-terminus of ApDGAT1/2b on DGAT activity and oil production in *S. cerevisiae*

**Fig. 7:**
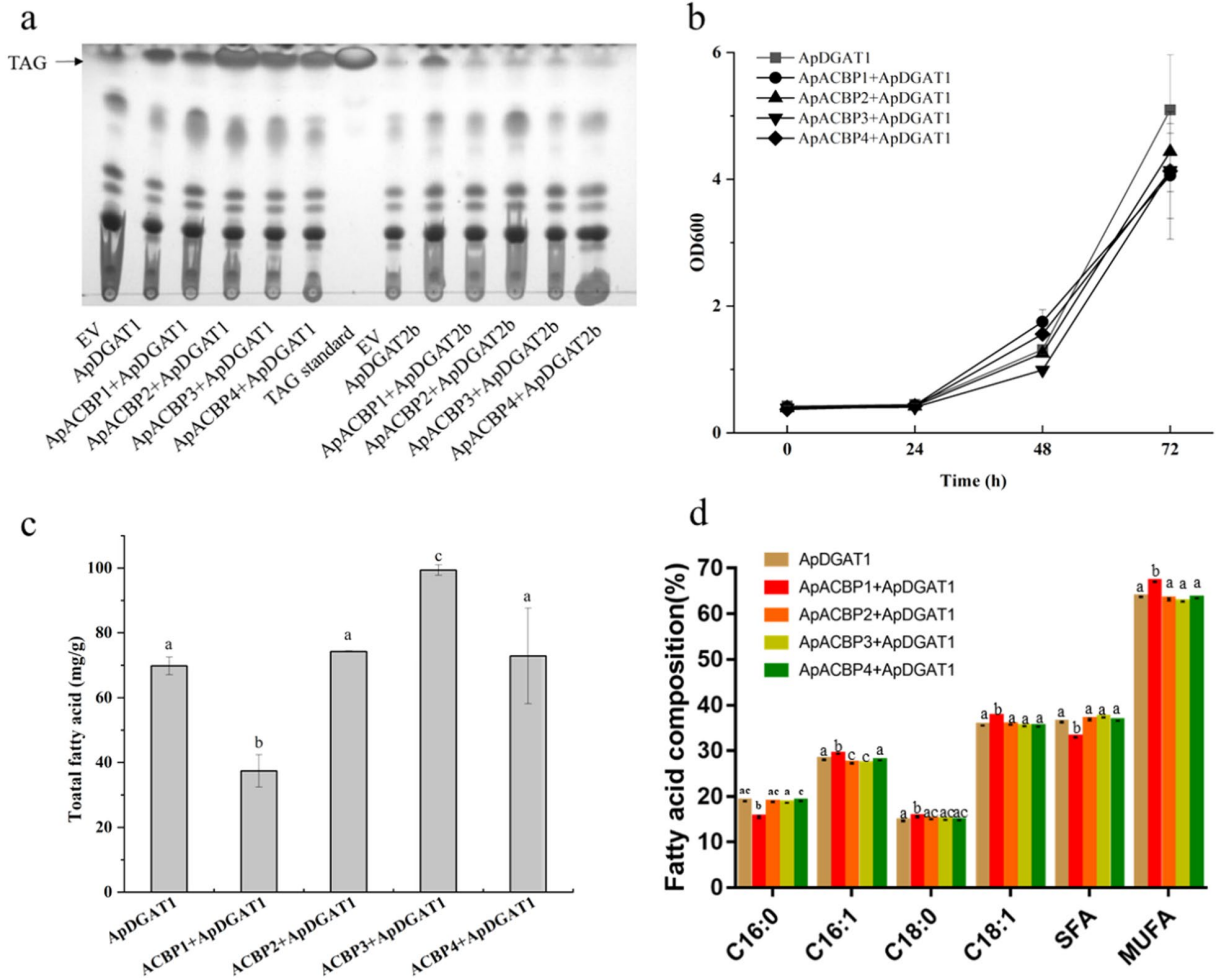
Functional study of ApACBPs + ApDGAT1/2b fusions in the *S. cerevisiae* H1246 system. **a** TLC analysis of the TAG synthesis of strains with ApACBPs + ApDGAT1 and ApACBPs + ApDGAT2b fusions. Comparison of **b** growth, **c** total fatty acid, **d** fatty acid composition between strains with ApACBPs + ApDGAT1 overexpressed. Data are mean ± SD. of three biological replicates. Different letters indicate significant differences at *p* < 0.05, as determined by one-way ANOVA with Tukey’s multiple comparisons test

Full-length enzymes of ApDGAT1/ApDGAT2b with four ApACBPs fused to their N-termini were individually introduced into the yeast strain of H1246 to explore the effects of structural modifications on DGAT activity and oil production. As shown in Fig. 7a, the fusion of four ApACBPs to ApDGAT1 did not affect the DGAT activity. In comparison, no TAG was produced when ApDGAT2b was fused with four ApACBPs. This result was consistent with the results as mentioned above that DGAT activity was sensitive to modifications in the N-termini of ApDGAT2b, suggesting that N-termini was essential to DGAT activity.

To further examine the effects of fusing ApACBPs to the N-terminus of ApDGAT1 on oil production, the total fatty acid content was quantified by gas chromatography. The overexpression of ApDGAT1 and ApACBPs + ApDGAT1 had no effects on the growth of yeast cells (Fig. 7b). The fusion of ApDGAT1 to ApACBP3 led to a significant increase of 42% in lipid accumulation in yeast compared with the expression of ApDGAT1 alone at 72 h (Fig. 7c). A slight decrease in unsaturated fatty acids of C16:1 and C18:1 content was observed, comparing to that of the original enzyme. In contrast, the overexpression of ApACBP1 + ApDGAT1 resulted in a 46% lower lipid content than that of the ApDGAT1 at the end of the culture period, but affected the content of palmitic acid (C16:0), palmitoleic acid (C16:1), stearic acid (C18:0) and oleic acid (C18:1Δcis9, C18:1) (Fig. 7d). Whereas, no changes in lipid production and fatty acid composition were observed on the yeast expressed with ApACBP2 + ApDGAT1 and ApACBP4 + ApDGAT1, compared with yeast overexpressed with ApDGAT1 and ApACBP2 + ApDGAT1 and ApACBP4 + ApDGAT1. Thus, a combination of ApACBP3 + ApDGAT1 with the potential to improve DGAT activity and increase oil content was successfully screened. The fusion of four ApACBPs to ApDGAT1 led to different enzyme activity levels, and the various mutants had different substrate preferences of fatty acids. These results indicated that four ApACBPs had different roles in lipid metabolism.

### Overexpression of ApDGATs boosted oleic acid accumulation and oil content, and ApACBP3 + ApDGAT1 further increased the content of ALA in the transgenic microalgae *C. reinhardtii* CC-5235

**Fig. 8:**
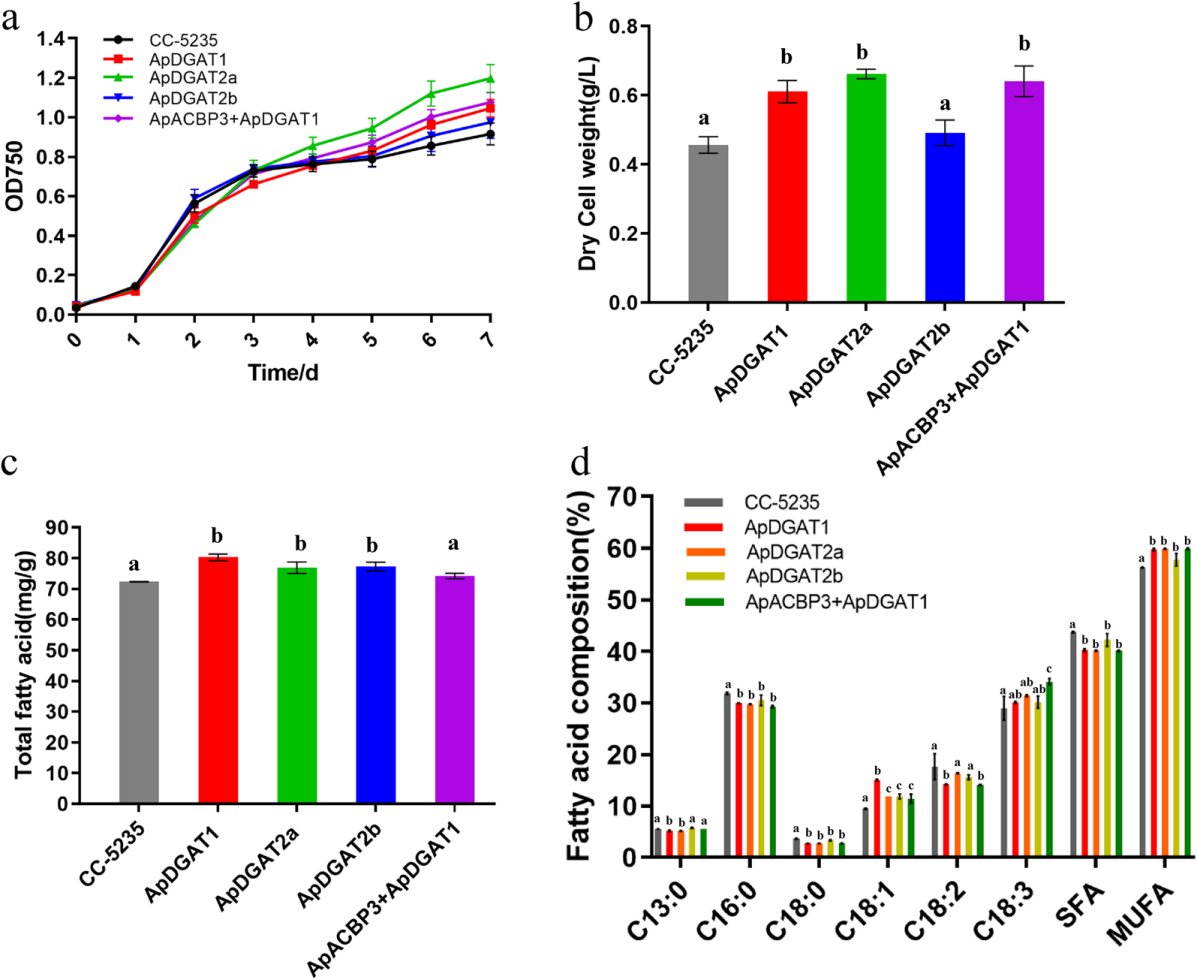
Phenotypes of *C. reinhardtii* CC-5235 transformants with ApDGATs and ApACBP3 + ApDGAT1 overexpressed. Growth (**a**), Dry cell weight (**b**), and Total fatty acid contents (**c**) of fatty acids in *C. reinhardtii* CC-5235 transformed with ApDGATs and ApACBP3 + ApDGAT1. **d** Relative abundance of fatty acids in *C. reinhardtii* CC-5235 transformed with ApDGATs and ApACBP3 + ApDGAT1. Data are mean ± SD. of three biological replicates. Different letters indicate significant differences at *p* < 0.05, as determined by one-way ANOVA with Tukey’s multiple comparisons test

To further explore the role and engineering potential of ApDGATs and ApACBP3 + ApDGAT1 fusion in modulating lipid synthesis in microalgae, ApDGATs and ApACBP3 + ApDGAT1 mutants were individually introduced into the model algal *C. reinhardtii* CC-5235(Additional file 1: Fig. S4). The strain with ApDGAT2b saw no differences in growth rate compared to the wild type, whereas the strains with ApDGAT1, ApDGAT2a, and ApACBP3 + ApDGAT1 exhibited increased growth during the stationary phase under normal conditions (Fig. 8a). Biomass yield of strains with ApDGAT1, ApDGAT2a and ApACBP3 + ApDGAT1 was significantly increased by approximately 34.0%, 45.2%, and 40.6% compared to that of WT (Fig. 8b), respectively. These findings indicated that overexpression of ApDGAT1, ApDGAT2a, and ApACBP3 + ApDGAT1 affected cell growth.

The lipid content in the wild type and transformant strains were shown in Fig. 8c. In both strains with ApDGATs overexpressed, the lipid content was significantly increased, especially in that with ApDGAT1, which increased by 10.9%. In contrast, the lipid content in ApACBP3 + ApDGAT1 was slightly increased, insignificant compared with that of the wild-type strain. Intriguingly, ApDGAT2a, which showed no DGAT activity in the functional complementation analysis in H1246, promoted the oil content and cell growth in *C. reinhardtii* CC-5235. It might be due to the differences in genetic traits of these two organisms. The transgenic effects of *C. reinhardtii* CC-5235 on fatty acid composition were investigated. According to the lipid composition analysis of three ApDGATs strains, the content of oleic acid (C18:1) was significantly increased by 59.2%, 24.7%, and 24.7%, accompanied by a reduction of SFAs (C16:0 and C18:0). In addition, strains with overexpressed ApDGAT1, ApDGAT2a and ApDGAT2b contained lower proportions of total saturated fatty acids and higher proportions of unsaturated fatty acids than wild-type cells (Fig. 8d). These results indicated that ApDGATs had a strong preference for unsaturated fatty acids (C18:1) over SFAs (C16:0 and C18:0). Interestingly, compared to the ApDGAT1 group, ApACBP3 + ApDGAT1 had a more significant impact on improving the ALA (C18:3) productivity as a 1.20-fold increase in ALA content was observed, suggesting that ApACBP3 might preferentially bound to C18:3-CoA. Thus ApACBP3 fusion to ApDGAT1 resulted in higher ALA accumulation in lipid synthesis.

### Potential of physical interaction of ApDGAT1 with itself, ApDGAT2b, and ApACBP1 facilitating the enrichment of oleic acid

**Fig. 9:**
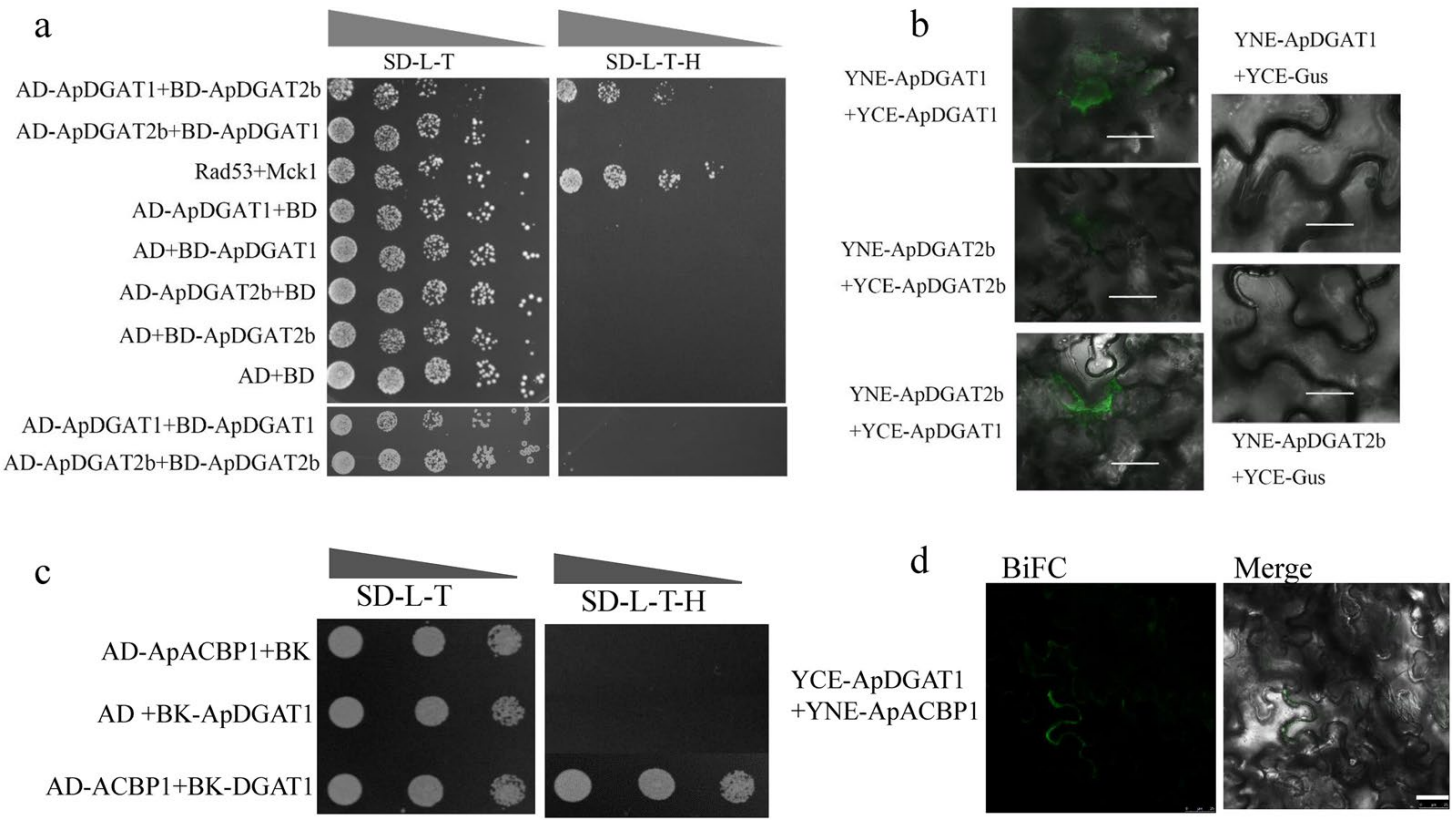
Possible physical interactions among ApDGATs and ApACBPs. Interactions between ApDGATs and ApACBPs in yeast using yeast two-hybrid (Y2H) (**a**, **c**) and in *N. benthamiana* leaves using a BiFC assay (**b**, **d**). The scale bar in **b** and **d** was 50 μm and 25 μm, respectively

Physical interactions between lipid biosynthetic enzymes, such as DGATs, have been detected in plants, mammals, and microalgae *C. zofingiensis* [22, 26–28].To explore and verify the potential protein − protein interactions between ApDGAT1, ApDGAT2b and ApACBPs, the methods of yeast two-hybrid (Y2H) screening and bimolecular fluorescence complementation (BiFC) assays were used. As shown in Fig. 9a, yeast strains transformed with AD-ApDGAT1/BD-ApDGAT2b showed the same growth rate as the positive control Rad53/Mck1 on SD-L–T–H, while strains transformed with AD-ApDGAT2b/BD-ApDGAT2b showed weaker growth in SD-L–T–H. These results indicated a strong interaction between ApDGAT1 and ApDGAT2b, but a weak self-association of ApDGAT2b. The BiFC experiments further confirmed that ApDGAT1 and ApDGAT2b could bind with themselves to form homologous dimers, and ApDGAT1 could also interact with ApDGAT2b in tobacco leaves (Fig. 9b). In addition, ApDGAT1 interacted with ApACBP1 in yeast and tobacco leaves (Fig. 9c, d). It should be noted that in the Y2H experiments (Fig. 9a), different bait and prey combinations of the same protein pairs led to different results. This discrepancy was also reported when the interactions between *Linum usitatissimum* PCAT2 and PDAT1, and *Arabidopsis* DGAT and LPCAT were tested. One possible reason was that the membrane-bound feature of these proteins could limit the orientation of the two termini of proteins and thus affect their interactions [28, 30]. It could be concluded that ApDGAT1 could physically interact with itself, DGAT2b, and ApACBP1, indicating that microalgae lipid metabolic enzymes (ApDGAT1, ApDGAT2b, and ApACBP1) might have been a part of a dynamic protein interactome that facilitated the enrichment of oleic acid of *A. protothecoides* UTEX 2341.

## Discussion

Previous studies reported that many microalgae contained one or two copies of DGAT1 and several copies of DGAT2 [46]. For example, *C. reinhardtii*, *Volvox carteri f. nagariensis*, *Coccomyxa subellipsoidea* and *Chlorella variabilis* had a single DGAT1 and an abundant of DGAT2 genes [46]. There were five copies of DGAT2 in *C. reinhardtii* and *Haematococcus pluvialis* [2, 47], eight copies of DGAT2 in *C. zofingiensis* and thirteen copies of DGAT2 in *Nannochloropsis oceanica* [10, 42]. These observations were consistent with our phylogenetic analysis. But the sequence analyses revealed that *A. protothecoides* UTEX 2341 contained one single DGAT1 and two DGAT2 genes, and the number of DGAT2 genes was smaller than that of the other reported microalgae. This result was consistent with the comparatively small genome size of *A. protothecoides*. For example, the genome sequencing results of *A. protothecoides* 0710 showed that it had the smallest genome reported among the sequenced chlorophytes, with a smaller number of genes and fewer multi-copy genes than its close relatives [40]. In addition, the functional analysis of ApDGATs showed that DGAT1 played a major role in TAG synthesis, which was consistent with the reported results of microalgae *C. zofingiensis* and *H. pluvialis* and plants[2, 42], indicating that DGAT1 family function was conserved in evolution. However, DGAT2 genes differed in many properties, such as mRNA expression profiles, DGAT activity, and substrate preferences. This phenomenon was ubiquitously observed in DGAT2 genes of microalgae, such as HpDGTT1, HpDGTT3, and HpDGTT4 of *H. pluvialis*, NoDGAT2J and NoDGAT2K of *N. oceanica,* and CzDGTT1 and CzDGTT2 of *C. zofingiensis* [2, 18, 42], suggesting that compared with DGAT1, the DGAT2 family was not conservative in function.

Different DGATs exhibited Acyl‑CoA substrate specificity and DAG substrate specificity. NoDGAT1A, for example, preferred saturated acyl-CoAs (C16:0 and C18:0), and contributed to incorporating more saturated fatty acids than the unsaturated ones into TAG in *N. oceanica* [36]. In addition, *C. zofingiensis* DGAT1A preferred the C16:0, C18:2 and C18:3, while *C. reinhardtii* DGTT2 preferred C18:0 and C18:1n9 [37, 44], indicating that different DGAT had different preferences. ApDGAT2a did not function as an acyltransferase in the yeast functional complementarity assay, its overexpression in *C. reinhardtii* CC-5235 promoted lipid accumulation. The functional failure of the ApDGAT2a in yeast might have been caused by the absence of a suitable substrate in yeast, such as C18:2 and C18:3. It was observed that the addition of C18:2 and C18:3 promoted TAG accumulation in *H. pluvialis* DGTT2 and *C. zofingiensis* DGAT2 [2, 20]. *H. pluvialis* DGTT2 was designated as a DGAT2 clade according to the phylogenetic analysis. However, its function analysis showed that it had lysophosphatidic acyltransferase (LPAAT) activity [2]. Therefore, it was hypothesized that ApDGAT2a might have played a role as a LPAAT based on its conserved domains, but further evidence was needed. It was also observed that overexpression of ApDGATs and ApDGAT1 + ApACBP3 had different effects on the profile of fatty acids in yeast and *C. reinhardtii* CC-5235. Saturated fatty acids (C16:0, C18:0) might be preferred in yeast (Figs. 3e, 4c, 7d), while unsaturated fatty acids, such as C18:1, might be favored in *C. reinhardtii* CC-5235 (Fig. 8d). Due to the relative small scale of biological duplication (triplicate), the accuracy of fatty acid profiles needed to be further analyzed in the future.

Moreover, previous phylogenetic analyses of DGATs showed that ApDGATs clustered together with the DGAT family of microalgae, and according to the functional verification results, DGATs in *C. reinhardtii* CC-5235 were closer to that of the original *A. protothecoides* UTEX 2341. These results indicated the importance of selecting the appropriate host for the functional verification of target genes. Currently, functional verification of DGATs mostly used *S. cerevisiae* as the host due to factors like its clear genetic background. However, there were limitations in expressing plant-derived functional genes in yeast systems due to the poor adaptability of genes to host strains and their different substrate preferences. Therefore, this study is of reference value for further studies on the functional genes of microalgae.

Distinct structure–function features of DGAT1 and DGAT2b were further investigated. DGAT1 was highly conserved and possessed a variable hydrophilic N-terminus, the different segments of which up-regulated or down-regulated the enzymatic activity [21]. The phylogenetic analysis of DGAT1 showed that the PH domain existed only in some microalgae. Moreover, it was located at the N-terminus of the variable hydrophobic region of DGAT1. Previous studies revealed that the PH domain was an essential structure for the TAG-synthesis capacity of *M. incise* DGAT1, which could promote the binding of this protein to the cell membrane to fully exert its enzymatic activity [44]. However, it was not essential for the ApDGAT1 activity of TAG synthesis. It was hypothesized that the PH domain of ApDGAT1 might have played a different role from that in MiDGAT1, such as the recruitment of other interacting proteins, which needed to be further verified. In contrast, the first 55 amino acids of the N terminus down-regulated the enzymatic activity, which was consistent with previous reports that the N-terminus of DGAT1 was variable and might have played an important role in enzyme regulation. Contrary to the stable structure–function of the ApDGAT1, the structure–function of the DGAT2 gene family varied in different species, such as *S. cerevisiae*, Murine, and *C. zofingiensis*. As for ApDGAT2b, truncation analysis showed that removing either the TMD1 or the entire N-terminus region led to complete enzyme inactivity in TAG synthesis. These results were much different from those of Murine DGAT2 and CzDGAT2s, which were not essential for DGAT activity [24]. Therefore, the DGAT2 gene family was not conservative, which might be due to the evolutionary adjustments specific to mammals, fungi, and plants.

Protein–protein interactions have been shown to play important physiological roles in various organisms, especially in recent studies on lipid biosynthetic enzymes. According to BiFC and Co-IP assays, AtDGAT1 and AtPDAT1 of *A. thaliana* could form a complex, which efficiently promoted TAG synthesis to meet special metabolic needs [48]. Flax DGAT2 could interact with LPCAT, PDCT, and itself to promote the full use of substrates [30]. In addition, mouse DGAT1 and DGAT2 existed in polymers that could adjust the number of active and inactive DGATs in response to changes in environmental conditions [22, 24]. Furthermore, it was found that ACBP2 probably interacted directly with lysophospholipase 2 in *A. thaliana,* thereby promoting lysophosphatidylcholine hydrolysis [49]. Interestingly, this study showed that ApDGAT1 interacted with not only itself and ApDGAT2b but also ApACBP1, further extending the protein–protein interaction of lipid biosynthetic enzymes to microalgae and proving that a dynamic protein interactome might exist in microalgae. Moreover, when ApDGAT1 and ApDGAT2b were overexpressed in *C. reinhardtii* CC-5235, the accumulation of oleic acid (C18:1) was significantly increased. A similar observation was also found when the ApACBP1 was fused to the N-terminus of ApDGAT1 in yeast (Fig. 7c). The significant accumulation of C18:1 in *C. reinhardtii* CC-5235 was consistent with results observed on fermented microalgae *A. protothecoides* UTEX 2341, and the relative content of oleic acid accounted for the highest proportion of total fatty acids, reaching up to 47.30% in normal fermentation culture of *A. protothecoides* UTEX 2341 [34]. It meant that ApDGATs and ApACBP1 might have played a potential role in oleic acid biosynthesis in *A. protothecoides* UTEX 2341. These results indicated that interactions between ApDGAT1, ApDGAT2b and ApACBP1 might facilitated the enrichment of oleic acid. Moreover, it would be of value in the exploration of the possible contribution of ApDGAT1, ApDGAT2b and ApACBP1 interactions to TAG biosynthesis and cell physiology in *A. protothecoides* UTEX 2341.

## Conclusions

This study characterized three DGATs and explored their structure–function importance using truncation mutagenesis in conjunction with enzyme assay. An improved ApDGAT1 variant was successfully screened by fusing different ACBPs to the N-terminus of full-length ApDGAT1/ApDGAT2b, and its engineering potential for lipid modulation in yeast and algae *C. reinhardtii* was explored. Furthermore, a dynamic protein interactome in microalgae lipid metabolic enzymes contributing to effective oleic acid biosynthesis was discovered. In conclusion, this study revealed a distinct structure–function relationship of DGATs in the *A. protothecoides* UTEX 2341, and uncovered a dynamic protein interactome in microalgae, which provided valuable information for the understanding of lipid metabolism.

## Supplementary Information

**Additional file 1: Fig. S1.** Domain analysis of ApDGATs proteins. **Fig. S2.** Transmembrane domain analysis of ApDGATs by TMHMM (V2.0, http://www.cbs.dtu.dk/services/TMHMM/). **Fig. S3.** PCR analysis of different truncated mutants of ApDGAT1 and ApDGAT2b from transgenic lines of *S. cerevisiae* H1246. **Fig. S4.** Phylogenetic analysis and functional motif analysis of ApACBPs. (a) Analysis of the protein domains of ApACBPs. (b) Analysis of the conservative motifs of ApACBPs. (c) Phylogenetic analysis of ApACBPs. **Fig. S5.** PCR analysis of ApDGATs and ApACBP3 + ApDGAT1 genes from transgenic lines of *C. reinhardtii*. (a, b) Amplification of the 700 bp fragment of the ApDGAT1 and ApDGAT2a gene. M, DNA 2000 + marker. (c) Amplification of the ApDGAT2b gene from the transformants. M, DNA 2000 + marker. (d) Amplification of the 300 bp fragment of the ApACBP3 + ApDGAT1 gene. M, DNA 5000 + marker. + , positive control; -, wild-type *C. reinhardtii*; 1–10, putative transformants.

**Additional file 2: Table S1.** QPCR primers used for quantitative analysis of the expression level of ApDGATs in *A. protothecoides* UTEX 2341. **Table S2.** Primers used for cloning of the full-length coding sequence of *A. protothecoides* ApDGATs and ApACBPs. **Table S3.** Proteins used for amino acid alignment of ApDGATs. **Table S4.** DGAT proteins used for the construction of the phylogenetic tree. **Table S5.** Primers used for cloning the truncated coding sequence of ApDGAT1 and ApDGAT2b. **Table S6.** Overview of putative DGATs and ACBPs cDNAs identified in the *A. protothecoides* genomic database. **Table S8**. Fusion expression primers of ApACBPs with ApDGAT1 and ApDGAT2b

## Data Availability

All relevant data can be found within the manuscript and its supporting materials. The GeneBank Accession Numbers of ApDGAT1, ApDGAT2a and ApDGAT2b were MT602552, MT602663, and MT602554.
