# Supplementary material for: Characterization of Auxenochlorella protothecoides acyltransferases and potential of their protein interactions to promote the enrichment of oleic acid
Source: Biotechnol Biofuels Bioprod. 2023 Apr 21;16:69. doi: 10.1186/s13068-023-02318-y (PMC10120206; doi:10.1186/s13068-023-02318-y)
Supplement: Supplementary file 1 — Additional file 1: Fig. S1. Domain analysis of ApDGATs proteins. Fig. S2. Transmembrane domain analysis of ApDGATs by TMHMM (V2.0, http://www.cbs.dtu.dk/services/TMHMM/). Fig. S3. PCR analysis of different truncated mutants of ApDGAT1 and ApDGAT2b from transgenic lines of S. cerevisiae H1246. Fig. S4. Phylogenetic analysis and functional motif analysis of ApACBPs. (a) Analysis of the protein domains of ApACBPs. (b) Analysis of the conservative motifs of ApACBPs. (c) Phylogenetic analysis of ApACBPs. Fig. S5. PCR analysis of ApDGATs and ApACBP3 + ApDGAT1 genes from transgenic lines of C. reinhardtii. (a, b) Amplification of the 700 bp fragment of the ApDGAT1 and ApDGAT2a gene. M, DNA 2000 + marker. (c) Amplification of the ApDGAT2b gene from the transformants. M, DNA 2000 + marker. (d) Amplification of the 300 bp fragment of the ApACBP3 + ApDGAT1 gene. M, DNA 5000 + marker. + , positive control; -, wild-type C. reinhardtii; 1–10, putative transformants. [file 13068_2023_2318_MOESM1_ESM.docx]

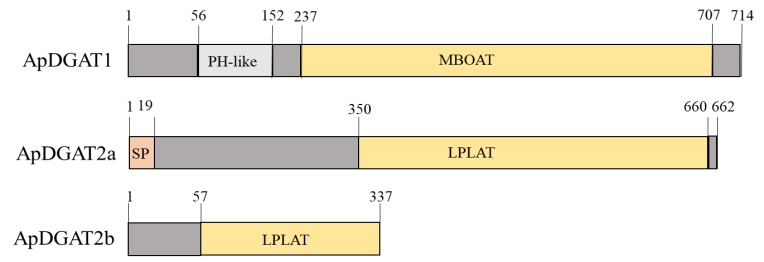


**Fig. S1** Conserved Domain analysis of three ApDGATs proteins.


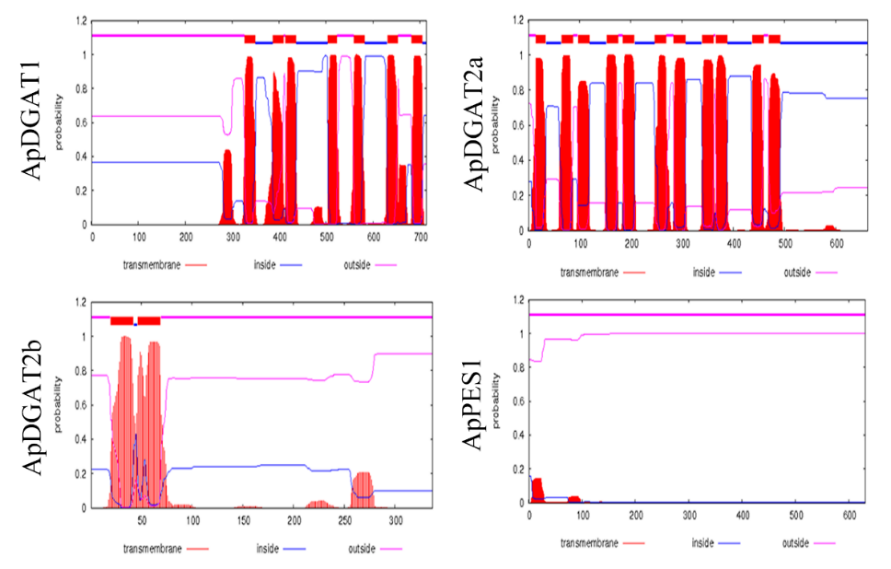


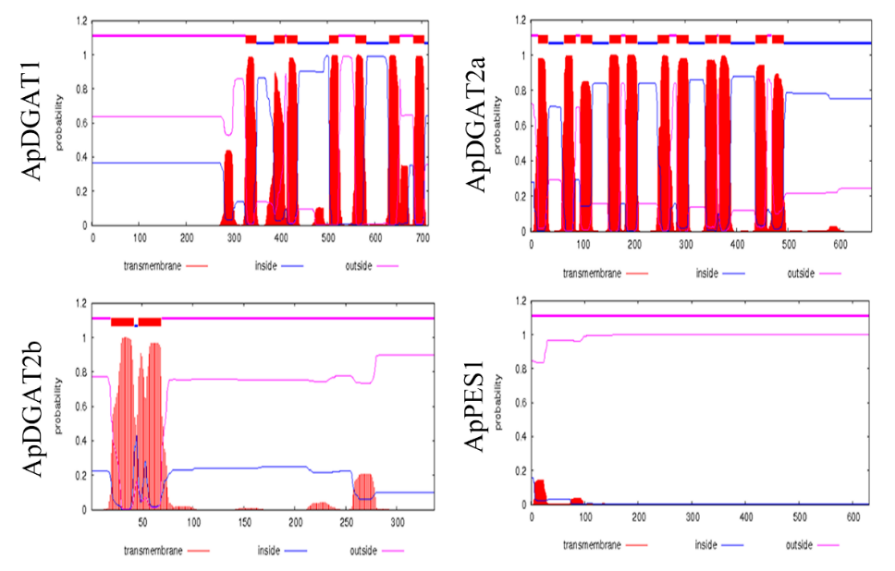


**Fig. S2** Analysis of transmembrane domains of three ApDGATs proteins.


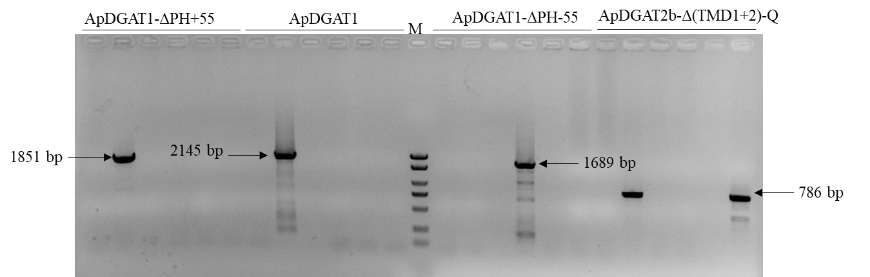


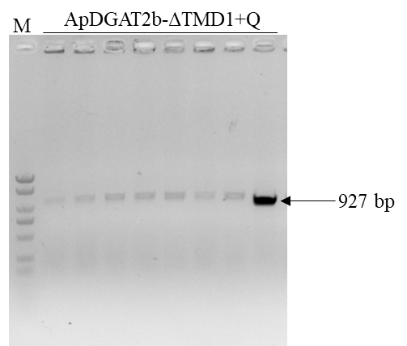

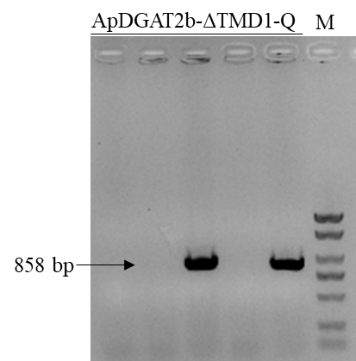


**Fig. S3** PCR analysis of different truncated mutants of ApDGAT1 and ApDGAT2b from transgenic lines of *S.cerevisiae* H1246. M, DNA 2000+ marker.


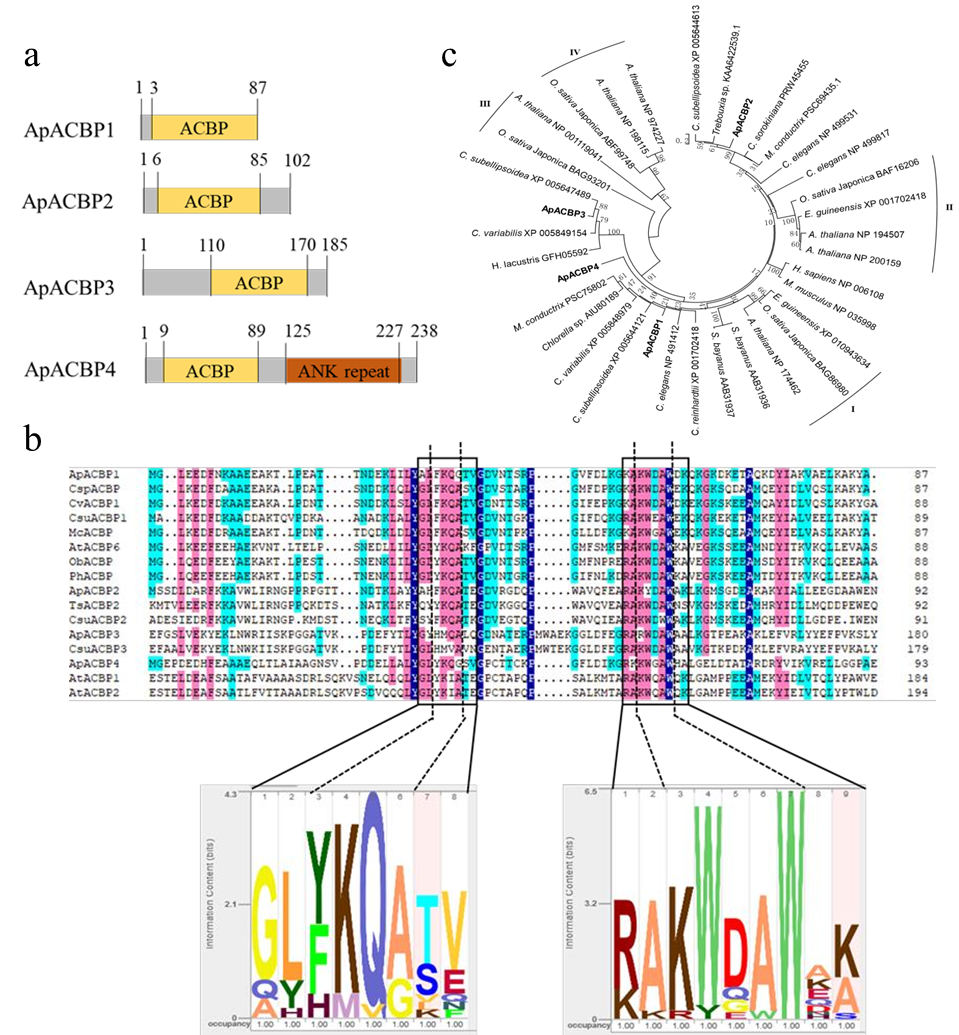


**Fig. S4** Phylogenetic analysis and functional motif analysis of ApACBPs. (a) Analysis of the protein domains of ApACBPs. (b) Analysis of the conservative motifs of ApACBPs. (c) Phylogenetic analysis of ApACBPs.


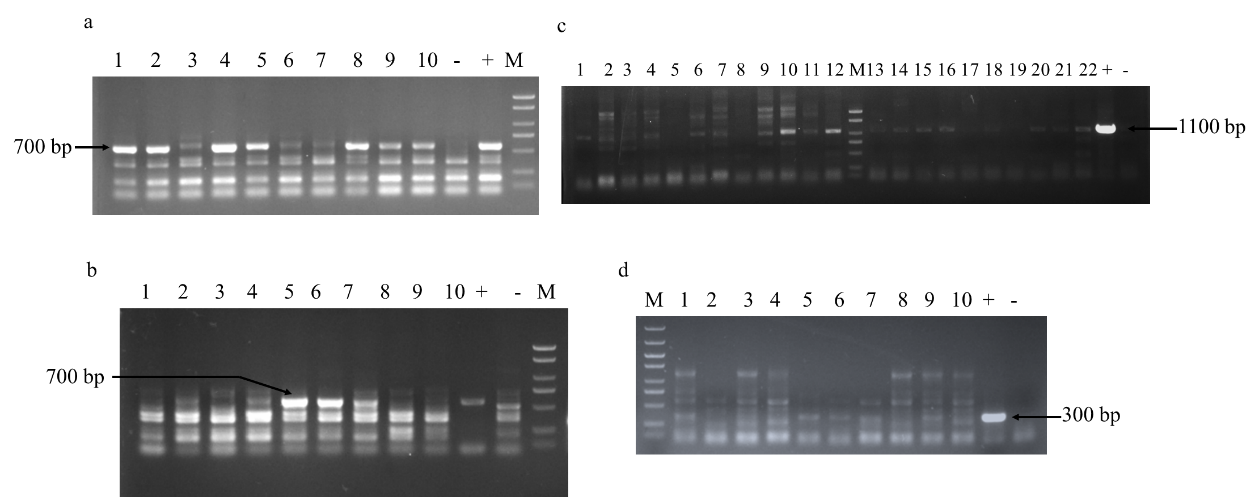


**Fig. S5** PCR analysis of ApDGATs and ACBP3+DGAT1 genes from transgenic lines of *C. reinhardtii*. (a, b) Amplification of the 700 bp fragment of the ApDGAT1 and ApDGAT2a gene. M, DNA 2000+ marker. (c) Amplification of the DGAT2b gene from the transformants. M, DNA 2000+ marker. (d) Amplification of the 300 bp fragment of the ACBP3+ApDGAT1 gene. M, DNA 5000+ marker. +, positive control; -, wild-type *C. reinhardtii*; 1–10, putative transformants.
