# Supplementary material for: Characterization of Auxenochlorella protothecoides acyltransferases and potential of their protein interactions to promote the enrichment of oleic acid
Source: Biotechnol Biofuels Bioprod. 2023 Apr 21;16:69. doi: 10.1186/s13068-023-02318-y (PMC10120206; doi:10.1186/s13068-023-02318-y)
Supplement: Supplementary file 2 — Additional file 2: Table S1. QPCR primers used for quantitative analysis of the expression level of ApDGATs in A. protothecoides UTEX 2341. Table S2. Primers used for cloning of the full-length coding sequence of A. protothecoides ApDGATs and ApACBPs. Table S3. Proteins used for amino acid alignment of ApDGATs. Table S4. DGAT proteins used for the construction of the phylogenetic tree. Table S5. Primers used for cloning the truncated coding sequence of ApDGAT1 and ApDGAT2b. Table S6. Overview of putative DGATs and ACBPs cDNAs identified in the A. protothecoides genomic database. Table S8. Fusion expression primers of ApACBPs with ApDGAT1 and ApDGAT2b [file 13068_2023_2318_MOESM2_ESM.docx]

**Table S1**. QPCR primers using for quantitative the expression level of ApDGATs in *A. protothecoides* UTEX 2341

| Gene Name | Length (bp) | Forward and reverse primer (5’-3’) |
| --- | --- | --- |
| ApDGAT1 | 80 | CGCATTGGCAACGCTATT CAGTCGTGGTAGTACAGCAT |
| ApDGAT2a | 98 | GTGGCGACCATCATCATC AAGGTGACAGCAATGTGAG |
| ApDGAT2b | 91 | GCTATGTGCCCATGCTCA GCTCTCCGATCACGATGT |

**Table S2**. Primers using for the cloning of full-length coding sequence of *A. protothecoides DGAT*s.

| Gene Name | Forward and reverse primer (5’-3’) | Connected Vector |
| --- | --- | --- |
| ApDGAT1 | ATGTCCAAAGAGAGGGTTTCG  CTATGCGCCATGCTCCTTCCT | pTOPO-blunt vector |
| ApDGAT2a | ATGCCCCCCGTGGACCCC  CTACTTGTCAACCAGGACGAACTT |  |
| ApDGAT2b | ATGGTCGAAACGGAACGGGGG  TCAGTACACTATGAGCTTGA |  |
| ApACBP1 | ATGGGTCTGGAGGAGGATTTCAAC  TCAGGCATACTTGGCCTTCAG |  |
| ApACBP2 | ATGTCGAGCGATCTGGACGCC  TCACGGATCTGGGTAGTCCTT |  |
| ApACBP3 | ATGACCATCGACGCTGGAACT  TCAGGCACGGGTGTCGGAGTA |  |
| ApACBP4 | ATGGGTGAGCCGGACGAG  TCAGGCTGGCTGCAGGCT |  |
| ApDGAT1 | CCCAAGCTTATGTCCAAAGAGAGGGTTTCG  ATTTGCGGCCGCCTATGCGCCATGCTCCTTCCT | pYES2/CT |
| ApDGAT2a | CCCAAGCTTATGCCCCCCGTGGACCCC  ATTTGCGGCCGCCTACTTGTCAACCAGGACGAACTT |  |
| ApDGAT2b | CCCAAGCTTATGGTCGAAACGGAACGGGGG  ATTTGCGGCCGCTCAGTACACTATGAGCTTGAG |  |
| ApDGAT1 | CCCAAGCTTATGTCCAAAGAGAGGGTTTCG  GACTAGTTGCGCCATGCTCCTTCCT | pSuper1300-GFP |
| ApDGAT2b | CCCAAGCTTATGGTCGAAACGGAACGGGGG  GGACTAGTGTACACTATGAGCTTGAGGGT |  |
| ApDGAT1 | GGAATTCCATATGATGTCCAAAGAGAGGGTTTCG  CGGGATCCCTATGCGCCATGCTCCTTCCT | pGADT7 |
| ApDGAT2b | GGAATTCCATATGATGGTCGAAACGGAACGGGGG  CGGGATCCTCAGTACACTATGAGCTTGA |  |
| ApDGAT1 | CGGAATTCATGTCCAAAGAGAGGGTTTCG  GCGTCGACCTATGCGCCATGCTCCTTCCT | pGBDT7 |
| ApDGAT2b | CGGAATTCATGGTCGAAACGGAACGGGGG  GCGTCGACTCAGTACACTATGAGCTTGA |  |
| ApDGAT1 | GCTCTAGAATGTCCAAAGAGAGGGTTTCG  CCCTCGAGTGCGCCATGCTCCTTCCT | pSPYNE  pSPYCE |
| ApDGAT2b | GCTCTAGAATGGTCGAAACGGAACGGGGG  CCCTCGAGGTACACTATGAGCTTGAGGGT |  |

Underlined sequences designate the restriction enzyme sites.

**Table S3.** DGAT proteins used for the construction of the phylogenetic tree

| Protein name | Source and Genbank Accession Number |
| --- | --- |
| ApDGAT1 | This study |
| AtDGAT1 | *Arabidopsis thaliana*, acc. No. AAF19262 |
| CvDGAT1 | *Chlorella variabilis*, acc. No.XP_005842809 |
| GmDGAT1 | *Glycine max*, acc. No. AAS78662 |
| OsDGAT1 | *Oryza sativa*, acc. No. AAW47581 |
| PpDGAT1 | *Pseudochlorella pringsheimii，*acc. No. KT779429 |
| PtDGAT1 | *Phaeodactylum tricornutum*, acc. No.XP_002177753 (partial) |
| TeDGAT1 | *Tetraselmis* sp, acc. No. JAC66181 |
| TmDGAT1 | *Tropaeolum majus*, acc. No. AAM03340 |
| TpDGAT1 | *Thalassiosira pseudonana*, acc. No.ADV58933 |
| ApDGAT2A | This study |
| ApDGAT2B | This study |
| AtDGAT2 | *Arabidopsis thaliana,* acc. No. NP_566952 |
| BtDGAT2A | *Bos taurus*, acc. No.DAA21853 |
| CeDGAT2A | *Caenorhabditis elegans*, acc. No.NP_505413 |
| CrDGAT2A | *Chlamydomonas reinhardtii*, acc. No. XP_001694904.1 |
| CrDGAT2B | *Chlamydomonas reinhardtii*, acc. No. XP_001693189.1 |
| CsDGAT2 | *Chlorella sorokiniana*, acc. No.PRW20576.1 |
| CsODGAT2 | *Coccomyxa* sp. Obi, acc. No.BBG28519.1 |
| CvDGAT2 | *Chlorella variabilis*, acc. No. EFN51306.1 |
| DrDGAT2 | *Danio rerio,* acc. No. NP_001025367 |
| EoDGAT2 | *Ettlia oleoabundans*, acc. No.AID16200.1 |
| GmDGAT2 | *Glycine max*, acc. No.ACU20344 |
| LiDGAT2.3 | *Lobosphaera incisa*，acc. No.AZI70899.1 |
| McDGAT2 | *Micractinium conductrix*, acc. No.PSC70219.1 |
| PpDGAT2 | *Physcomitrella patens*，acc. No.XP_024360450.1 |
| RsDGTA2 | *Raphidocelis subcapitata*, acc. No.GBF89369.1 |
| PtDGAT2A | *P.tricornutum*, acc. No. JX469835 |
| VfDGAT2 | *Vernicia fordii*, acc. No. ABC94473 |
| AhDGAT3 | *Arachis hipogaea*, acc. No.AAX62735 |
| AtDGAT3 | *A. thaliana*, acc. No.AAK06873 |
| OsDGAT3 | *O. sativa*, acc. No.AAS98422 |

**Table S4.** DGAT1 proteins used for the construction of the phylogenetic tree

| Protein name | Source | Accession Number | Protein length (aa) |
| --- | --- | --- | --- |
| ApDGAT1 | *Auxenochlorella protothecoides* | This study | 714 |
| CeDGAT1 | *Chlorella elliptica* | KT779429.1 | 713 |
| PtDGAT1 | [*Phaeodactylum tricornutum*](https://www.ncbi.nlm.nih.gov/protein/ADY76581.1/) | ADY76581.1 | 564 |
| NoDGAT1A | *Nannochloropsis oceanica* | ASL69957.1 | 437 |
| CzDGAT1A | *Chromochloris zofingiensis* | QBG05553.1 | 742 |
| CvuDGAT1 | *Chlorella vulgaris* | ALP13865.1 | 460 |
| TpDGAT1 | *Thalassiosira pseudonana* | XP_002287215.1 | 432 |
| CvDGAT1 | *Chlorella variabilis* | XP_005842809.1 | 445 |
| TeDGAT1 | *Tetraselmis* sp. GSL018 | JAC66181.1 | 704 |
| TgDGAT1 | *Toxoplasma gondii* | AAP94209.1 | 540 |
| ZmDGAT1 | *Zea mays* | ABV91586.1 | 494 |
| VvDGAT1 | *Vitis vinifera* | XP_002279345.1 | 518 |
| AtDGAT1 | *Arabidopsis thaliana* | NP_179535.1 | 520 |
| BoDGAT1 | *Brassica oleracea* | XM_013751131.1 | 510 |
| BtDGAT1 | *Bos Taurus* | AAL49962.1 | 489 |
| EaDGAT1 | *Euonymus alatus* | AAV31083.1 | 507 |
| EpDGAT1 | *Echium pitardii* | ACO55634.1 | 473 |
| GmDGAT1a | *Glycine max* | NP_001237289.1 | 498 |
| GmDGAT1b | *Glycine max* | NP_001237684.2 | 504 |
| HaDGAT1 | *Helianthus annuus* | ACD67882.1 | 479 |
| HsDGAT1 | *Homo sapiens* | NP_036211.2 | 488 |
| JcDGAT1 | *Jatropha curcas* | ABB84383.1 | 521 |
| LjDGAT1 | *Lotus japonicus* | AAW51456.1 | 511 |
| BnDGAT1 | *Brassica napus* | AFM31259.1 | 501 |
| BjDGAT1 | *Brassica juncea* | AAY40784 | 503 |
| TmDGAT1 | *Tropaeolum majus* | AAM03340 | 518 |
| OsDGAT1 | *Oryza sativa* | AAW47581 | 538 |
| NtDGAT1 | *Nicotiana tabacum* | AAF19345.1 | 532 |
| MmDGAT1 | *Mus musculus* | NP_034176.1 | 498 |
| MtGAT1 | *Medicago truncatula* | XP_003595231.1 | 539 |
| OeDGAT1 | *Olea europaea* | AAS01606.1 | 532 |
| PfDGAT1 | *Perilla frutescens* | AAG23696.1 | 534 |
| RcDGAT1 | *Ricinus communis* | NP_001310663.1 | 521 |
| SsDGAT1 | *Sus scrofa* | NP_999216.1 | 489 |
| VfDGAT1 | *Vernicia fordii* | ABC94471.1 | 526 |
| VgDGAT1 | *Vernonia galamensis* | ABV21945.1 | 523 |
| LiDGAT1 | *Lobosphaera incisa* | AYE66854 | 745 |
| McDGAT1 | *Micractinium conductrix* | PSC71143 | 724 |
| CsDGAT1 | *Chlorella sorokiniana* | PRW60121 | 728 |
| CsODGTA1 | *Coccomyxa* sp. Obi | BBG28515 | 768 |
| GpDGAT1 | *Gonium pectorale* | KXZ55102 | 801 |
| CeuDGAT1 | *Chlamydomonas eustigma* | GAX78660 | 1038 |
| HlDGAT1 | *Haematococcus lacustris* | QOY44183 | 828 |

**Table S5**. Primers using for the cloning of truncated coding sequence of *ApDGAT1 and ApDGAT2b.*

| Forward and reverse primer (5’-3’) | Gene name |
| --- | --- |
| CCCAAGCTTATGTCCAAAGAGAGGGTTTCG | ApDGAT1-ΔPH+55 |
| CGCCGGCACCCGGCGCGTTCCTGAGTCGCGCCCTG |  |
| ACCTCCCAGGGCGCGACTCAGGAACGCGCCGGGTGCCGG |  |
| GCTCTAGATGCGCCATGCTCCTTCCT |  |
| CCCAAGCTTATGGAACGCGCCGGGTGCCGG | ApDGAT1-ΔPH-55 |
| GCTCTAGATGCGCCATGCTCCTTCCT |  |
| CCCAAGCTTATGGTCGAAACGGAACGGGGG  CACGGTGCCCAGCTTGTGCCCGACATGGCCCAGGAG | ApDGAT2b-ΔTMD1+Q |
| GGCCATGTCGGGCACAAGCTGGGCACCGTGAGCCTG  GCTCTAGAGTACACTATGAGCTTGAGGGT |  |
| CCCAAGCTTATGAAGCTGGGCACCGTGAGCCTG  GCTCTAGAGTACACTATGAGCTTGAGGGT | ApDGAT2b-ΔTMD1-Q |
| CCCAAGCTTATGAAATTCATGACCCGGTATCTG  GCTCTAGAGTACACTATGAGCTTGAGGGT | ApDGAT2b-ΔTMD(1+2)-Q |

**Table S6.** Overview of putative DGATs and ACBPs cDNAs identified in the *A.protothecoides* genomic database.

| Genes | Gene ID | cDNA length (bp) | Protein length | Molecular mass (KDa) | | Isoelectric point |
| --- | --- | --- | --- | --- | --- | --- |
| ApDGAT1 | F751_6502 | 2145 | 714 aa | | 79.57 | 9.57 |
| ApDGAT2a | F751_1386 | 1989 | 662 aa | | 72.27 | 9.42 |
| ApDGAT2b | F751_0071 | 1014 | 337 aa | | 37.97 | 9.56 |
| ApACBP1 | F751_1235 | 264 | 87 aa | | 9.7 | 6.28 |
| ApACBP2 | F751_2578 | 309 | 102 aa | | 11.48 | 6.08 |
| ApACBP3 | F751_4226 | 558 | 185 aa | | 20.30 | 7.80 |
| ApACBP4 | F751_1055 | 717 | 238 aa | | 24.89 | 4.53 |

**Table S8.** Fusion expression primers of ApACBPs with ApDGAT1 and ApDGAT2b

| Primer name | Primer sequence | Fragment |
| --- | --- | --- |
| ACBP-1235-HindIII-F | CCCAAGCTTATGGGTCTGGAGGAGGATTTCAAC | ApACBP1+ApDGAT1 |
| 1235-DGAT1-OV-R | CTCTCTTTGGACATGGCATACTTGGCCTTCAGCTC |  |
| 1235-DGAT1-OV-F | AGGCCAAGTATGCCATGTCCAAAGAGAGGGTTTCG |  |
| H1246-AT1B-XbaI-NZ | GCTCTAGATGCGCCATGCTCCTTCCT |  |
| ACBP-2578-HindIII-F | CCCAAGCTTATGTCGAGCGATCTGGACGCC | ApACBP2+ApDGAT1 |
| 2578+1-OV-R-2 | AACCCTCTCTTTGGACATCGGATCTGGGTAGTCCTTGAG |  |
| 2578+1-OV-F-2 | AAGGACTACCCAGATCCGATGTCCAAAGAGAGGGTTTCG |  |
| H1246-AT1B-XbaI-NZ | GCTCTAGATGCGCCATGCTCCTTCCT |  |
| ACBP-4226-HindIII-F | CCCAAGCTTATGACCATCGACGCTGGAACT | ApACBP3+ApDGAT1 |
| 4226+1-OV-R-2 | AACCCTCTCTTTGGACATGGCACGGGTGTCGGAGTAC |  |
| 4226+1-OV-F-2 | TACTCCGACACCCGTGCCATGTCCAAAGAGAGGGTTTCG |  |
| H1246-AT1B-XbaI-NZ | GCTCTAGATGCGCCATGCTCCTTCCT |  |
| ACBP-1055-HindIII-F | CCCAAGCTTATGGGTGAGCCGGACGAGGAT | ApACBP4+ApDGAT1 |
| 1055+1-OV-R-2 | AACCCTCTCTTTGGACATGGCTGGCTGCAGGCTCCAGGC |  |
| 1055+1-OV-F-2 | TGGAGCCTGCAGCCAGCCATGTCCAAAGAGAGGGTTTCG |  |
| H1246-AT1B-XbaI-NZ | GCTCTAGATGCGCCATGCTCCTTCCT |  |
| ACBP-1235-HindIII-F | CCCAAGCTTATGGGTCTGGAGGAGGATTTCAAC | ApACBP1+ApDGAT2b |
| 1235-DGAT2b-OV-R | TCCGTTTCGACCATGGCATACTTGGCCTTCAGCTC |  |
| 1235-DGAT2b-OV-F | AGGCCAAGTATGCCATGGTCGAAACGGAACGGGGG |  |
| H1246-AT2-XbaI-NZ | GCTCTAGAGTACACTATGAGCTTGAGGGT |  |
| ACBP-2578-HindIII-F | CCCAAGCTTATGTCGAGCGATCTGGACGCC | ApACBP2+ApDGAT2b |
| 2578-DGAT2b-OV-R | GTTCCGTTTCGACCATCGGATCTGGGTAGTCCTTGAGTGC |  |
| 2578-DGAT2b-OV-F | ACTACCCAGATCCGATGGTCGAAACGGAACGGGGG |  |
| H1246-AT2-XbaI-NZ | GCTCTAGAGTACACTATGAGCTTGAGGGT |  |
| ACBP-4226-HindIII-F | CCCAAGCTTATGACCATCGACGCTGGAACT | ApACBP3+ApDGAT2b |
| 4226-DGAT2b-OV-R | TCCGTTTCGACCATGGCACGGGTGTCGGAGTACAA |  |
| 4226-DGAT2b-OV-F | CCGACACCCGTGCCATGGTCGAAACGGAACGGGGG |  |
| H1246-AT2-XbaI-NZ | GCTCTAGAGTACACTATGAGCTTGAGGGT |  |
| ACBP-1055-HindIII-F | CCCAAGCTTATGGGTGAGCCGGACGAGGAT | ApACBP4+ApDGAT2b |
| 1055+2b-OV-R-2 | CCGTTCCGTTTCGACCATGGCTGGCTGCAGGCTCCAGGC |  |
| 1055+2b-OV-F-2 | TGGAGCCTGCAGCCAGCCATGGTCGAAACGGAACGGGGG |  |
| H1246-AT2-XbaI-NZ | GCTCTAGAGTACACTATGAGCTTGAGGGT |  |
